# Chemical Stimulants and Stressors Impact the Outcome of Virus Infection and Immune Gene Expression in Honey Bees (*Apis mellifera*)

**DOI:** 10.3389/fimmu.2021.747848

**Published:** 2021-10-28

**Authors:** Fenali Parekh, Katie F. Daughenbaugh, Michelle L. Flenniken

**Affiliations:** ^1^ Department of Microbiology and Immunology, Montana State University, Bozeman, MT, United States; ^2^ Department of Plant Sciences and Plant Pathology, Montana State University, Bozeman, MT, United States; ^3^ Pollinator Health Center, Montana State University, Bozeman, MT, United States

**Keywords:** honey bee, *Apis mellifera*, insect antiviral defense, honey bee viruses, deformed wing virus, thymol, fumagillin, clothianidin

## Abstract

Western honey bees (*Apis mellifera*) are ecologically, agriculturally, and economically important plant pollinators. High average annual losses of honey bee colonies in the US have been partially attributed to agrochemical exposure and virus infections. To examine the potential negative synergistic impacts of agrochemical exposure and virus infection, as well as the potential promise of phytochemicals to ameliorate the impact of pathogenic infections on honey bees, we infected bees with a panel of viruses (i.e., Flock House virus, deformed wing virus, or Sindbis virus) and exposed to one of three chemical compounds. Specifically, honey bees were fed sucrose syrup containing: (1) thyme oil, a phytochemical and putative immune stimulant, (2) fumagillin, a beekeeper applied fungicide, or (3) clothianidin, a grower-applied insecticide. We determined that virus abundance was lower in honey bees fed 0.16 ppb thyme oil augmented sucrose syrup, compared to bees fed sucrose syrup alone. Parallel analysis of honey bee gene expression revealed that honey bees fed thyme oil augmented sucrose syrup had higher expression of key RNAi genes (*argonaute-2* and *dicer-like*), antimicrobial peptide expressing genes (*abaecin* and *hymenoptaecin)*, and *vitellogenin*, a putative honey bee health and age indicator, compared to bees fed only sucrose syrup. Virus abundance was higher in bees fed fumagillin (25 ppm or 75 ppm) or 1 ppb clothianidin containing sucrose syrup relative to levels in bees fed only sucrose syrup. Whereas, honey bees fed 10 ppb clothianidin had lower virus levels, likely because consuming a near lethal dose of insecticide made them poor hosts for virus infection. The negative impact of fumagillin and clothianidin on honey bee health was indicated by the lower expression of *argonaute-2*, *dicer-like*, *abaecin*, and *hymenoptaecin*, and *vitellogenin*. Together, these results indicate that chemical stimulants and stressors impact the outcome of virus infection and immune gene expression in honey bees.

## 1 Introduction

Honey bees (*Apis mellifera*) are important pollinators of fruit, nut, and vegetable crops that make up a large proportion of the human diet (1–3). The value of pollination services in the United States is $14.6 billion annually (4). Maintaining the honey bee pollination force has been challenging in many parts of the world, including the US, where annual colony losses averaged 38% from 2008 to 2018 (5–10). A combination of biotic and abiotic factors impact colony health including lack of quality nutritional resources, exposure to beekeeper and/or grower applied chemicals, parasites, and pathogens [reviewed in (11–13)]. The ectoparasitic mite, *Varroa destructor*, is responsible for a large percentage of colony deaths in the US and Europe (14–16) reviewed in (17). *Varroa* mites feed on the bee fat bodies and hemolymph, kill brood, and vector viruses, including deformed wing virus within and between colonies (17–21). Honey bee pathogens, including bacteria, microsporidia, fungi, trypanosomatids, and viruses also contribute to honey bee colony deaths.

The largest category of honey bee infecting pathogens are positive-sense single-stranded RNA (+ssRNA) viruses [reviewed in (22, 23)]. Honey bee infecting viruses include members of the *Dicistroviridae* family [e.g., Israeli acute paralysis virus (IAPV), Kashmir bee virus (KBV), acute bee paralysis virus (ABPV), and black queen cell virus (BQCV)], the *Iflaviridae* family [e.g., deformed wing virus (DWV), sacbrood virus (SBV)], chronic bee paralysis virus (CBPV), and the Lake Sinai viruses (LSVs) [reviewed in (22, 24)]. DWV is one of the most well-characterized, globally distributed bee-infecting viruses (25, 26). In temperate climates, the prevalence and/or abundance of DWV typically increases throughout the beekeeping season (15, 27–29). High levels of DWV have been associated with honey bee colony deaths (15, 30–34). Honey bees infected with DWV during development may have wing deformities, whereas DWV infection in adult bees may result in asymptomatic infections or symptomatic infections characterized by shortened abdomen, cuticle discoloration, and reduced lifespan (15, 25, 35, 36). While other factors may influence the severity of virus infections, the effectiveness of honey bee antiviral defense mechanisms is the greatest determinant of the outcome of viral infection.

Honey bee immune defense mechanisms include NF-κB mediated Toll and Imd (Immune Deficiency), Jak/STAT (Janus Kinase/Signal Transducer and Activator of Transcription), JNK (c-Jun N-terminal kinase), MAPK (Mitogen-Activated Protein Kinases), and RNA interference (RNAi) pathways [reviewed in (22, 37)]. The antiviral role of NF-κB mediated Toll and Imd pathways in response to specific viruses has, in part, been determined by examining the expression of antimicrobial peptide encoding genes, including *abaecin* and *hymenoptaecin*, in conjunction with quantifying virus abundance (37–41). RNA viruses produce long dsRNA molecules as they replicate within cells. These viral associated molecule patterns (VAMPS) are recognized as non-self and trigger antiviral responses. In honey bees and bumble bees dsRNA-mediated antiviral responses include both sequence-specific RNA interference (RNAi) and a non-sequence specific virus reducing response (42–47). RNAi mediated antiviral defense is initiated by the recognition of long double stranded RNAs (dsRNAs) by endoribonuclease, Dicer, which cleaves these dsRNAs into 21-23 nucleotide long small interfering RNAs (siRNAs) (48). These siRNAs are incorporated into the RNA-induced silencing complex (RISC), the non-target binding passenger strand is released, and the remaining strand in the RISC targets cognate RNAs, including viral genomes and transcripts, for Argonaute mediated cleavage, thereby lowering viral abundance (49–55). Although, the functions of these proteins have been best-characterized in other organisms, including *Drosophila melanogaster (* 52, 55, 56 *)*, the antiviral role of the RNAi pathway has been demonstrated *in vivo* in adult honey bees and larvae. Transcriptome analyses of virus-infected honey bees revealed that the expression of *dicer-like* and *ago2* was increased in response to SINV-GFP or IAPV infection, but not in response to DWV (34, 42, 43, 45, 57–59).

In addition to the immune pathways described above, the heat shock response is involved in antiviral defense in fruit flies and honey bees (60, 61). The heat shock response pathway is induced by various stressors including heat-stress (62). In the context of viral infection, the role of the heat shock response must be assessed for individual host-virus pairs, since heat shock proteins can both positively negatively affect viral replication (62–65). In honey bees, the expression of several heat shock protein encoding genes including *heat shock protein 90 (hsp90)*, and *heat shock 70-kDa protein cognate 4* (*hsc70-4)* are induced by SINV-GFP infection and/or heat-shock, and the expression of *protein lethal (* 2)*essential for life-like (pl2)*, which encodes a protein with an Hsp20-domain, is modulated in honey bees infected with several viruses (i.e., IAPV, DWV, and SINV-GFP) (43, 57, 61, 66). Similar to the heat shock response genes, another honey bee gene that is involved in multiple functions including immunity, nutrition, stress resistance, behavioral development, ageing, oxidative stress, and longevity is *vitellogenin* (*vg*) (67–76). Therefore, *vg* expression is often utilized as a proxy for either or both honey bee age and health status [reviewed in (77)] (78–80).

Studies aimed at better characterizing the impact of viruses on honey bee health and the mechanisms of honey bee antiviral defense have involved the use of naturally-infected bees, which often have varying levels of infections, or bees experimentally inoculated with honey bee viruses (e.g., DWV, IAPV, or mixed virus stocks) or model viruses (43, 45, 61, 81). In this study, we carried out experiments using a panel of viruses. Specifically, we utilized DWV, and two model viruses [i.e., Flock House virus (FHV) and Sindbis virus tagged with green fluorescent protein (SINV-GFP)]. While it is important to carry out studies with known honey bee infecting viruses, honey bee virus preparations obtained from pupae may include co-purifying viruses, as well as other proteins, and they are not a standardized source of infectious material. The recent development of infectious clones of honey bee viruses is promising, but they have not been utilized for studies in laboratories beyond those in which they were developed (82–84).The use of model viruses circumvents the problems associated with analyzing data obtained from studies impacted by pre-existing/confounding infections, enables preparation of standardized viral stocks from cultured cells, and also facilitates comparative studies across different insect species. Flock House virus (FHV) naturally infects grass grubs (*Costelytra zealandica*), and is extensively utilized model virus that infects a wide range of other insects including mosquitoes, fruit flies, tsetse flies, and honey bees (85–91). FHV has evolved a mechanism to counter the host immune response; it encodes for protein B2, a viral suppressor of RNAi [reviewed in (92)]. SINV-GFP is another well-characterized model virus that has been extensively utilized to investigate antiviral defense mechanisms in a wide range of insects including fruit flies, mosquitos, and honey bees (42, 43, 61, 93). SINV-GFP is easily trackable both visually using microscopy, and at the protein level *via* Western blot analyses. Unlike FHV, Sindbis virus does not encode a viral suppressor of RNAi (94–96). Overall, the use of a panel of viruses to examine the impact of abiotic factors on the honey bee antiviral response and the outcome of virus infection is important to ensure that results are robust and potentially generalizable.

The honey bee immune system, and in turn the outcome of virus infections in individual bees is influenced by nutritional status and environmental factors (97–100). Honey bees gather pollen and nectar from flowering plants which provision the colony with nutrients including proteins, lipids, carbohydrates, vitamins, and phytochemicals (101). In addition, honey bees gather plant resins, which are the primary component of propolis (102). Recent research efforts have focused on interaction of phytochemicals and pathogens, since plant-derived compounds can either be toxic or medicinal depending on the administered dose (103). There is precedence in the literature that honey bee diets supplemented with phytochemicals increase longevity and prime the immune system by increasing the expression of antimicrobial peptide encoding genes, for example, in honey bees fed *p*-coumaric acid, the expression of antimicrobial peptides *defensin* and *abaecin* was two-fold higher compared to control bees (104–107). For the studies described herein, we investigated the impact of phytochemical containing thyme oil on the outcome of honey bee virus infections. Honey bees forage on common thyme plants (*Thymus vulgaris*), which produce thymol, a phytochemical that has shown promise in reducing DWV loads in honey bees (104). Thymol, a terpene found in pollen and nectar of *Thymus vulgaris*, is a major component of thyme oil. *T. vulgaris* nectar contains 5.2 - 8.2 ppm thymol, while thymol concentrations in bee-collected pollen samples range between 0.0263 to 55.8 ppm (108–110). Thymol has also been detected in honey (i.e., 0.27 ppm, 4-weeks post colony treatment with 8 grams per week for 4 weeks) (111). Thyme oil contains 10% - 64% thymol depending on the plant species, geographical sources, and harvest season, which may affect the volatile composition of the plant (112–118). Thymol is widely used as an acaricide and is an active ingredient in commercial *Varroa* control formulations such as Apiguard^®^ and ApiLife VAR^®^, which contain 25% and 74% thymol respectively (119, 120). Thymol treatments, ranging from 0.16 ppb - 120 ppm, have also shown promise in reducing levels of honey bee pathogens including *Crithidia bombi, Nosema ceranae*, a microsporidial/fungal pathogen, and DWV (104, 121–124). Previous research by Palmer-Young et al., determined that newly emerged honey bees that were fed a mixture DWV and 0.16 ppb thymol and released back in the colony, had 26-fold decrease in DWV levels 7 days post infection compared to bees exposed to DWV (104). However, in parallel studies the natural occurring DWV-levels in young bees that were maintained in laboratory and fed thymol (0.16 ppb) for 10 days had similar DWV levels to untreated bees. This study also determined that a higher concentration of thymol (16 ppm) was toxic to honey bees. Overall, the results described by Palmer-Young et al., were intriguing, but somewhat difficult to interpret due to the lack of quantification of DWV inoculum, which was hemolymph obtained from symptomatic bees; the high degree of variation in DWV abundance in individual bees (i.e., ΔCt ranged from -20 to 12); and the results describing similar DWV levels in virus-inoculated bees and control bees that were not fed thymol, indicating a high level of pre-existing DWV abundance in the honey bees utilized for their studies (104).

In addition to phytochemicals, honey bees are exposed to neonicotinoid insecticides, fungicides, acaricides, and herbicides (125, 126). Neonicotinoids, including clothianidin, are utilized by growers to reduce the number of insects including aphids, whiteflies, leafhoppers, and planthoppers that damage crops (e.g., canola, corn, canola, cotton, soybeans) (127). Neonicotinoids are neurotoxins that disrupt the insect nervous system by irreversibly binding to nicotinic acetylcholine receptors that transmit nerve signals. This prevents signal transduction from the neurotransmitter acetylcholine, leading to paralysis and death (128, 129). Although honey bees are not the target insect, clothianidin is highly toxic to honey bees at doses near the median lethal dose (LD_50_) of 3 ppb for oral exposure and 22 ppb for contact exposure (130–134). Clothianidin concentrations of up to 2.6 ppb have been detected in pollen samples and concentrations ranging from 1 to 14 ppb, with most ranging between 0.3 to 5.4 ppb, in the nectar of treated crops (133, 135–140). In addition to potential lethal impact of clothianidin, sublethal doses have been shown to negatively affect honey bee immunity, grooming and hygienic behavior, neural gene expression, flight activity and homing behavior (141–152). Furthermore, honey bees exposed to pesticides including chlorpyrifos, thiamethoxam, thiacloprid, and clothianidin have greater viral loads, indicating that chemical exposure in conjunction with virus infection have a negative synergistic impact (153–157). Higher virus abundance in individual bees is in part due to dampened immune responses (154). For example, young bees exposed to clothianidin had greater DWV loads due to negative modulation of the NF- κB pathway (154).

Honey bee colonies are also to exposed chemicals utilized as in-hive treatments against honey bee pathogens, including *Nosema ceranae* and *Nosema apis. Nosema* infections are treated at the colony level with fumagillin dicyclohexyl ammonium (i.e., Fumagilin-B^®^), a compound derived from *Aspergillus fumigatus* (158–160). Beekeepers typically treat nosemosis with the manufacturer’s recommended concentration of 25 ppm fumagillin, albeit field studies have recorded the use of higher concentrations (161–164). In fact, detectable levels of fumagillin residues (0.002 ppm- 0.066 ppm) persist inside the hives after treatment (159, 165). While fumagillin is generally considered safe for honey bee colonies, and that potential negative impacts may be negligible in comparison to nosemosis, reports of higher mortality in honey bee queens and workers fed fumagillin containing sucrose syrup led to the recommendations that bees should not be treated during the foraging season, and that treatments should be restricted to one time in the spring and fall (166–168).

To better understand the impact of a phytochemical (i.e., thymol) and agrochemicals, including a grower-applied insecticide (i.e., clothianidin) and a beekeeper applied fungicide (i.e., fumagillin), on the outcome of virus infection in honey bees, we carried out laboratory-based experiments on individual virus-infected honey bees that were fed chemical containing diets. For these studies, we utilized a panel of RNA viruses, including two model viruses FHV and SINV-GFP, and one honey bee virus, DWV. We hypothesized that thymol would act as an immunostimulant and reduce virus infection levels. In contrast, we hypothesized that virus-infected honey bees exposed to fumagillin or clothianidin would harbor more severe virus infections as a result of immunosuppressive effects. We determined that virus-infected honey bees fed thyme oil augmented sucrose syrup harbored lower virus levels, likely due to greater expression of key immune genes including *dcr-like, ago2, abaecin, hymenoptaecin*, and *vitellogenin*. Conversely, we determined that fumagillin and clothianidin exposure had a negative impact on honey bee immunocompetence with reduced expression of key immune genes including *dcr-like, ago2, abaecin, hymenoptaecin*, and *vitellogenin*, which resulted in higher virus abundance in chemical-fed bees than virus levels in bees fed only sucrose syrup. Together our results indicate that thyme oil acts as an immune stimulant and thus reduces viral burden, whereas other chemicals (i.e., fumagillin or clothianidin) negatively impact honey bee immune gene expression, and in turn result in more severe virus infections.

## 2 Results

### 2.1 Impact of Chemicals on Virus Infections in Honey Bees

#### 2.1.1 Virus Abundance Reduced in Honey Bees Fed Thyme Oil Augmented Sucrose Syrup

To further investigate the potential of thyme oil augmented diets to reduce viral burden, aged-matched adult bees were infected with FHV, SINV-GFP or DWV. Viruses were administered *via* injection, which mimicked *Varroa destructor* mite-mediated virus transmission and ensured that individual bees received equivalent doses. In these assays, virus-inoculated honey bees were fed 0.16 ppb thyme oil (~ 60 ppb thymol) augmented sucrose syrup and virus abundance was assessed at 72 hpi in three independent experiments. The 72 hpi timepoint was selected since previous studies documented readily detectable levels of disseminated virus (i.e., SINV-GFP and DWV) and corresponding host immune gene expression changes at that time point (42, 43, 57, 61, 66). To ensure that 72 hpi was also appropriate time to assess FHV infection levels, honey bees were inoculated with FHV (3.5 x 10^8^ FHV RNA copies per bee), and virus abundance was quantified by RT-qPCR at 6, 48, 72, and 96 hpi (**Supplemental Figure S1** and **Supplemental Table S2**). FHV abundance increased from 0 hpi to 72 hpi and decreased from 72 hpi to 96 hpi. Since FHV abundance peaked at 72 hpi, this timepoint was selected to assay for FHV infection levels.

**Figure 1:**
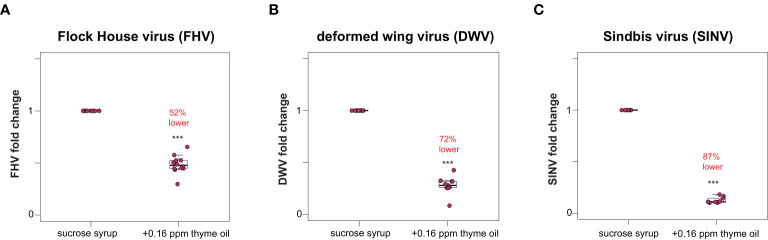
Lower virus abundance in honey bees fed thyme oil augmented sucrose syrup. Virus abundance in individual honey bees that were either fed sucrose syrup only or sucrose syrup augmented with 0.16 ppb thyme oil was assessed at 72 h post-infection by qPCR (n = 9-12 per treatment group) and the relative virus abundance is presented as ranked fold-changes. Together, these data illustrate that virus-infected bees fed thyme oil augmented sucrose syrup harbored less virus than virus-infected bees fed only sucrose syrup. **(A)** Flock House virus (FHV)-infected bees fed sucrose syrup augmented with thyme oil (0.16 ppb) had 52% less FHV (0.48 mean fold change) than bees fed sucrose syrup alone (*p* = 2.1 x 10^-5^). **(B)** In deformed wing virus (DWV)-infected bees, virus abundance was 72% less in bees fed sucrose syrup augmented with thyme oil (0.28 mean fold change) compared to bees fed only sucrose syrup (*p* = 0.00016). **(C)** Sindbis virus (SINV)-infected bees fed thyme oil augmented sucrose syrup had 87% less SINV (0.13 mean fold change) than bees fed sucrose syrup. Data were analyzed by a pairwise Wilcoxon Rank Sums with a Benjamini–Hochberg correction for multiple comparisons. Asterisks indicate a significant difference in virus abundance; significance levels: ***p < 0.0005. This figure includes results from one representative biological replicate for each virus (i.e., rep1). The data for all three biological replicates are presented in **Supplemental Figure S2** and raw data are in **Supplemental Tables S3**, **S4**.

Honey bees fed sucrose syrup augmented with thyme oil harbored less virus (i.e., FHV, DWV, SINV-GFP) compared to virus-infected bees fed sucrose syrup only (**Figure 1** and **Supplemental Figure S2**). Specifically, the total FHV abundance was 52% lower in honey bees fed thyme oil (0.16 ppb) augmented sucrose syrup compared to bees fed sucrose syrup only (n = 10 bees per treatment) (*p* < 0.001, Wilcoxon Rank Sums test with a Benjamini–Hochberg correction for multiple comparisons) (**Figure 1A**). Similar levels of reduction were observed in two additional experimental replicates with 48% and 35% reduction in FHV loads respectively (**Supplemental Figure S2**, *p* < 0.001). Similarly, DWV abundance was 72% lower in DWV-infected bees fed thyme oil (0.16 ppb) augmented sucrose syrup compared to bees fed sucrose syrup only (**Figure 1B**, *p* < 0.001). DWV abundance in two additional biological replicates was reduced by 48% and 25% in DWV-infected bees fed thyme oil augmented sucrose syrup compared to bees fed sucrose syrup only (**Supplemental Figure S2**, *p* < 0.01). Lastly, SINV-infected bees fed thyme oil augmented sucrose syrup had 87% less virus relative to SINV-infected bees fed sucrose syrup only (**Figure 1C**, *p* < 0.001). In two additional biological replicates of this experiment, SINV levels were reduced by 49% and 42% in SINV-infected bees fed thyme oil augmented sucrose syrup relative to bees fed sucrose syrup only (**Supplemental Figure S2**, *p* < 0.001). Together these results demonstrate that virus abundance was lower when virus-infected bees were fed 0.16 ppb thyme oil containing sucrose syrup compared to levels in bees fed non-augmented syrup and suggest that this dose of thyme oil is beneficial to honey bees.

#### 2.1.2 Greater Virus Abundance in Honey Bees Fed Fumagillin or Clothianidin Containing Sucrose Syrup

**Figure 2:**
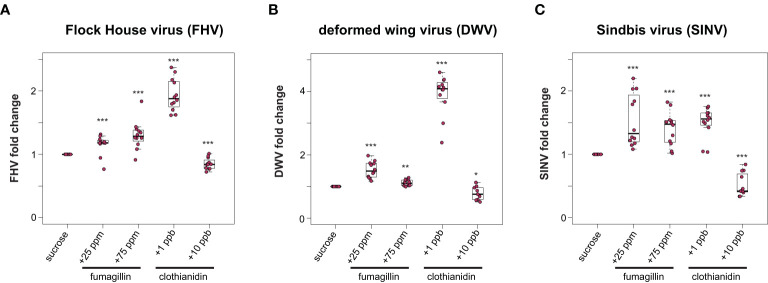
Honey bees fed sucrose syrup containing fumagillin or clothianidin had greater virus levels than bees feed only sucrose syrup. Virus abundance in individual honey bees that were either fed sucrose syrup only or fungicide or insecticide containing sucrose syrup was assessed at 72 h post-infection by qPCR (n = 9-12 per treatment group) and the relative virus abundance is presented as ranked fold-changes. Data from virus infected honey bees fed sucrose syrup mixed with fumagillin (25 ppm or 75 ppm) or clothianidin (1 ppb or 10 ppb) illustrate that bees fed sucrose syrup containing 25 ppm fumagillin, 75 ppm fumagillin, and 1 ppb clothianidin harbored more virus than virus-infected bees fed sucrose syrup only, whereas bees fed sucrose syrup containing 10 ppb clothianidin had lower virus levels **(A)** Flock House virus (FHV)-infected bees fed 25 ppm fumagillin (*p* = 0.006), 75 ppm fumagillin (*p* = 0.0009) or 1 ppb clothianidin (*p* = 8.2 x 10^-5^) containing sucrose syrup had greater virus levels than bees fed sucrose only syrup. Whereas, FHV-infected bees fed sucrose syrup containing 10 ppb clothianidin had lower virus levels than bees fed only sucrose syrup (*p* = 0.0009). **(B)** Deformed wing virus (DWV)-infected bees fed sucrose syrup containing 25 ppm fumagillin (*p* = 8.2 x 10^-5^), 75 ppm fumagillin (*p* = 0.0022) or 1 ppb clothianidin (*p* = 8.2 x 10^-5^) harbored more virus than DWV-infected bees fed only sucrose syrup. DWV-infected bees fed 10 ppb clothianidin containing sucrose syrup had less virus compared to bees fed sucrose syrup (*p* = 0.04). **(C)** Sindbis virus (SINV) abundance was higher in bees fed sucrose syrup containing 25 ppm fumagillin (*p* = 6.2 x 10^-5^), 75 ppm fumagillin (*p* = 6.2 x 10^-5^) or 1 ppb clothianidin (*p* = 6.2 x 10^-5^) compared to SINV-infected bees fed only sucrose syrup. In contrast, SINV-infected bees fed sucrose syrup containing 10 ppb clothianidin had lower levels of virus infection than bees fed sucrose only syrup (*p* = 6.8 x 10^-5^). Data were analyzed by a pairwise Wilcoxon Rank Sums with a Benjamini–Hochberg correction for multiple comparisons. Asterisks indicate a significant change in virus abundance; significance levels: *p < 0.05; **p < 0.005; ***p < 0.0005. This figure includes results from one representative biological replicate for each virus (i.e., rep3). The data for all three biological replicates are presented in **Supplemental Figure S3**. Raw data are included in **Supplemental Tables S5**, **S6**.

To examine the potential impact of other chemicals, including a beekeeper applied fungicide and a commonly used neonicotinoid insecticide on the outcome of virus infections, virus-infected honey bees were fed sucrose syrup containing fumagillin and clothianidin. Specifically, virus-infected honey bees were fed sucrose syrup containing 25 ppm or 75 ppm fumagillin, or a sublethal (1 ppb) or a near lethal dose (10 ppb) of clothianidin, and virus abundance was quantified at 72 hpi. We hypothesized that virus abundance would be greater in bees exposed to virus infection and chemical exposure. We determined that, in general, virus abundance was greater in honey bees that were exposed to chemicals in their diet. Specifically, FHV abundance in bees fed sucrose syrup containing 25 ppm or 75 ppm fumagillin was 15% (*p* = 0.006) and 30% higher (*p* < 0.001), respectively (**Figure 2A**). FHV-infected bees fed clothianidin (1 ppb) containing sucrose syrup had 94% more FHV relative to sucrose syrup fed bees (*p* < 0.001), whereas unexpectedly virus abundance was reduced by 15% in FHV-infected bees fed sucrose syrup containing 10 ppb clothianidin relative to FHV-infected bees fed sucrose syrup only (*p* < 0.001) (**Figure 2A**). Similarly, in DWV-infected bees, virus abundance was 53% higher in bees fed sucrose syrup containing 25 ppm fumagillin (*p* < 0.001), 11% higher in bees fed 75 ppm fumagillin sucrose syrup (*p* < 0.01), and 291% higher in bees fed 1 ppb clothianidin containing sucrose syrup (*p* < 0.001) (**Figure 2B**). DWV abundance was 21% lower in DWV-infected bees fed 10 ppb clothianidin containing sucrose syrup compared to DWV-infected bees fed sucrose syrup only (*p* = 0.04) (**Figure 2B**). Honey bees infected with SINV and fed 25 ppm fumagillin containing sucrose syrup harbored 53% more virus (*p* < 0.001), SINV-infected bees fed 75 ppm fumagillin containing sucrose syrup harbored 41% more virus (*p* < 0.001), while SINV infected bees fed 1 ppb clothianidin containing sucrose syrup had 50% more virus relative to sucrose only (*p* < 0.001). SINV-infected bees fed 10 ppb clothianidin containing sucrose syrup harbored 48% less SINV compared to bees fed sucrose syrup only (*p* < 0.001) (**Figure 2C**). Data obtained from two additional biological replicates resulted in similar changes in virus abundance when virus-infected bees (i.e., FHV, DWV, or SINV-GFP) were fed sucrose syrup containing fumagillin or clothianidin (**Supplemental Figure S3**, *p* < 0.05). Together these data indicate that virus-infected bees fed recommended (25 ppm) or higher (75 ppm) doses of fumagillin or 1 ppb clothianidin had greater viral loads, whereas bees fed 10 ppb clothianidin in sucrose syrup *ad libitum* had slightly lower virus levels at 72 hpi. This may be indicative of the poor overall health status of the host to support virus replication.

### 2.2 Impact of Chemicals on Honey Bee Immune Gene Expression

#### 2.2.1 Expression of Key RNAi Genes Is Higher in Thyme Oil Fed Bees, Reduced in Fumagillin and Clothianidin Fed Honey Bees

**Figure 3:**
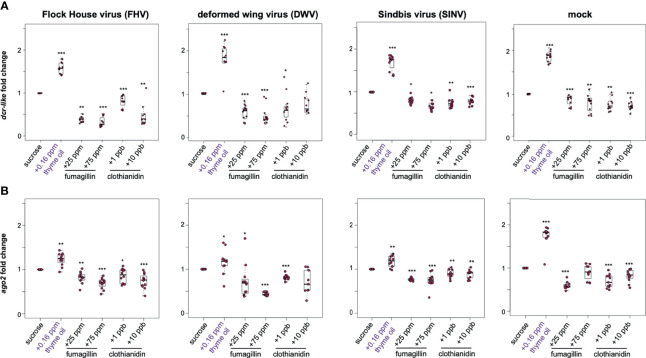
Expression of key RNAi genes was higher in honey bees fed thyme oil augmented sucrose syrup and lower in bees fed sucrose syrup containing fumagillin or clothianidin. Expression of *dicer-like* and *argonaute-2* in honey bees that were either mock- or virus-infected (i.e., FHV, DWV, SINV) and fed sucrose syrup only or syrup containing either thyme oil (0.16 ppb), fumagillin (25 ppm or 75 ppm), or clothianidin (1 ppb or 10 ppb) was assessed by qPCR. The ΔΔCt method with normalization to *rpl8* in mock or virus-infected bees fed sucrose syrup diet was utilized to determine the relative gene expression. **(A)** *Dicer-like (dcr-like)* expression was higher in virus-infected (i.e., FHV, DWV, SINV) and mock-infected bees fed thyme oil containing sucrose syrup, and lower in bees fed sucrose syrup containing fumagillin (25 ppm or 75 ppm) or clothianidin (1 ppb or 10 ppb) relative to bees fed sucrose syrup. **(B)** *Argonaute-*2 (*ago2)* expression was higher in virus-infected (i.e., FHV, DWV, SINV) or mock-infected bees fed thyme oil augmented sucrose syrup and reduced in bees fed diets containing fumagillin (25 ppm or 75 ppm) or clothianidin (1 ppb or 10 ppb), except in mock-infected bees fed 75 ppm fumagillin, *ago2* expression was similar to expression levels in bees fed sucrose only. Data were analyzed by a pairwise Wilcoxon Rank Sums with a Benjamini–Hochberg correction for multiple comparisons. Asterisks indicate a significant change in gene expression compared to sucrose only control; significance levels: *p < 0.05; **p < 0.005; ***p < 0.0005. This figure shows representative biological replicate for each gene (i.e., rep2 for *dcr-like* and rep3 for *ago2*). The data for all three biological replicates are presented in **Supplemental Figures S4**, **S5**. Raw data are included in **Supplemental Tables S7–S10**.

To investigate whether the changes in virus abundance in honey bees fed sucrose syrup containing additives were reflective of modulations in the expression of key RNAi genes, the expression of *argonaute-2 (ago2)* and *dicer-like (dcr)* were assessed at 72 hpi. In general, the expression of *dcr-like* and *ago2* were higher in virus-infected honey bees fed thyme oil containing sucrose syrup, and modestly reduced in virus-infected honey bees fed fumagillin or clothianidin containing sucrose syrup (**Figure 3**). Specifically, FHV-infected bees fed thyme oil exhibited 1.57 fold (*p* < 0.001) higher *dcr-like* expression and 1.23 fold higher *ago2* expression, compared to virus-infected bees fed sucrose syrup only (*p* = 0.001). Whereas FHV-infected bees fed sucrose syrup containing either 25 ppm or 75 ppm fumagillin exhibited reduced expression of *dcr-like* (i.e., 0.40 fold, *p* = 0.006, and 0.34 fold, *p* < 0.001, respectively) and *ago2* (i.e., 0.82 fold, *p* = 0.002 and 0.69 fold, *p* < 0.001, respectively). Similarly, FHV-infected bees fed clothianidin containing sucrose syrup expressed less *dcr-like* (i.e., 1 ppb - 0.81 fold, *p* < 0.001 and 10 ppb - 0.49 fold, *p* = 0.0031) and *ago2* (i.e., 1 ppb - 0.86 fold, *p* = 0.023 and 10 ppb - 0.74 fold, *p* < 0.01) (**Figure 3**). These results were consistent in two additional biological replicates of this experiment (**Supplemental Figure S4**, *p* < 0.05) except, in rep2, *ago2* expression in FHV-infected bees fed 1 ppb clothianidin was not reduced.

DWV-infected bees fed thyme oil (0.16 ppb) augmented sucrose syrup had 1.66 fold higher *dcr-like* expression relative to DWV-infected bees fed sucrose syrup only (*p* < 0.001). However, *dcr-like* expression in DWV-infected bees fed fumagillin was reduced (i.e., 25 ppm - 0.58 fold, *p* < 0.001 and 75 ppm - 0.49 fold, *p* < 0.001). A reduction in *dcr-like* expression was also observed in DWV-infected bees fed 1 ppb clothianidin (0.67 fold, *p* = 0.04), but *dcr-like* expression levels were not changed in bees fed 10 ppb clothianidin containing sucrose syrup (**Figure 3A**). The expression of *ago2* in DWV-infected bees fed thyme oil containing sucrose followed a similar trend with 1.16 fold higher expression (*p* = 0.035), and reduced expression in bees fed sucrose syrup containing 25 ppm fumagillin (0.72 fold, *p* = 0.024), 75 ppm fumagillin (0.43 fold, *p* < 0.001), and 1 ppb clothianidin (0.81 fold, *p* < 0.001), whereas *ago2* expression was not changed in DWV-infected bees fed sucrose syrup containing 10 ppb clothianidin (**Figure 3B**). These results were consistent in two additional biological replicates of this experiment (**Supplemental Figures S4, S5**, *p* < 0.05).

Comparably, SINV-infected bees fed thyme oil augmented sucrose syrup had higher levels of *dcr-like* expression than SINV-infected bees fed sucrose syrup only (i.e., 1.68 fold, *p* < 0.001) (**Figure 3A**). Whereas the combination of virus infection and chemical exposure resulted in reduced *dcr-like* expression (i.e., in SINV-infected bees fed 25 ppm fumagillin (0.81 fold, *p* = 0.014), 75 ppm fumagillin (0.66 fold, *p* = 0.018), 1 ppb clothianidin (0.74 fold, *p* < 0.01), and 10 ppb clothianidin containing sucrose syrup (0.79 fold, *p* < 0.001) compared to SINV-infected bees fed sucrose syrup only) (**Figure 3A**). Likewise, SINV-infected bees fed thyme oil containing sucrose syrup had 1.17 fold higher *ago2* expression (*p* = 0.002), whereas expression was slightly lower in bees fed sucrose syrup containing 25 ppm fumagillin 0.77 fold, (*p* < 0.001), 75 ppm fumagillin (0.75 fold, *p* < 0.001), 1 ppb clothianidin (0.9 fold, *p* < 0.01), and 10 ppb clothianidin (0.89 fold, *p* = 0.002) (**Figure 3B**). The majority of these results were consistent in two additional biological replicates of this experiment (**Supplemental Figures S4**, **S5**, *p* < 0.05) except *dcr-like* expression in SINV-infected bees fed 10 ppb clothianidin in rep3, and *ago2* expression in SINV-infected bees fed 25 ppm fumagillin in rep2 were similar to expression levels in virus-infected bees fed only sucrose syrup (*p* > 0.05). Together these results demonstrate that the expression of *dcr-like* and *ago2* were greater in virus-infected honey bees fed thyme oil (0.16 ppb) containing sucrose syrup, whereas expression of these genes was modestly reduced in virus-infected bees fed pesticide containing sucrose syrup.

To disentangle the gene expression results obtained from honey bees simultaneously impacted by two stressors (i.e., virus-infection and oral exposure to chemicals), gene expression was measured in non-virus infected honey bees (i.e., mock-infected). These analyses determined that the expression of both *dcr-like* and *ago2* were higher in thyme oil fed bees by 1.86 fold and 1.75 fold higher respectively (*p* < 0.001) (**Figure 3**). Whereas the expression of *dcr-like* and *ago2* in bees fed fumagillin or clothianidin was reduced, albeit to a lesser degree compared to the increase observed with thyme oil (**Figure 3**). Specifically, mock-infected bees fed fumagillin in sucrose at 25 ppm and 75 ppm concentrations had 0.77 fold (*p* < 0.001) and 0.77 fold lower *dcr-like* expression levels (*p* < 0.001), and 0.59 fold (*p* < 0.001), and no appreciable change in *ago2* expression, respectively. Likewise, mock-infected bees fed clothianidin in sucrose syrup at 1 ppb and 10 ppb concentrations had 0.75 fold (*p* < 0.01) and 0.72 fold (*p* < 0.001) *dcr-like* expression levels, and 0.69 fold (*p* < 0.001) and 0.81 fold (*p* < 0.001) *ago2* expression levels, respectively.

#### 2.2.2 Expression of Heat Shock Protein Encoding Genes Is Higher in Thyme Oil, Fumagillin, and Clothianidin Fed Honey Bees

**Figure 4:**
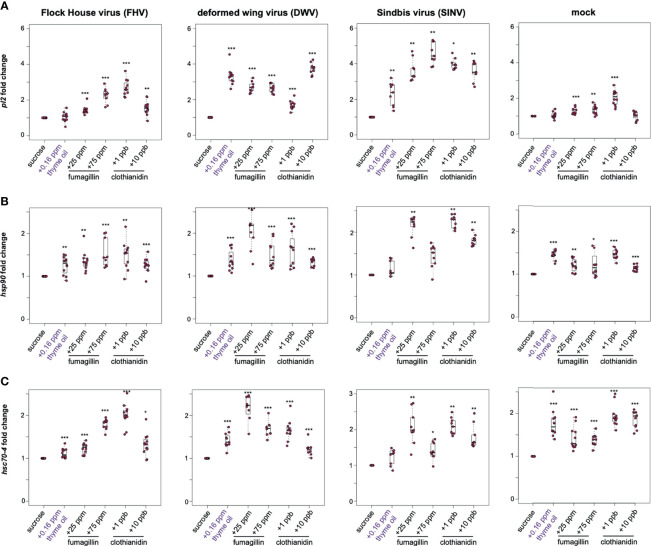
Expression of heat shock protein encoding genes was higher in honey bees fed sucrose syrup containing thyme oil, fumagillin, or clothianidin. The relative expression of three genes encoding heat shock proteins (*pl2, hsp90, hsc70-4*) was assessed using qPCR in mock or virus-infected bees fed sucrose syrup only or sucrose syrup containing additivities (i.e., thyme oil, fumagillin, or clothianidin). The ΔΔCt method with normalization to *rpl8* in mock or virus-infected bees fed sucrose syrup diet was utilized to determine the relative gene expression. **(A)** *Protein lethal(2)essential for life-like (pl2)* expression in virus-infected bees (i.e., FHV, DWV, SINV) fed sucrose syrup containing thyme oil, fumagillin (25 ppm or 75 ppm) or clothianidin (1 ppb or 10 ppb) was higher compared to expression in bees fed sucrose only. In mock-infected bees, *pl2* expression in bees fed sucrose syrup containing stimulant (0.16 ppb thyme oil) or 10 ppb clothianidin was similar to expression levels in bees fed sucrose only, whereas *pl2* expression was higher in bees fed sucrose syrup containing fumagillin (25 ppm or 75 ppm) or 1 ppb clothianidin. **(B)** *Heat shock protein 90 (hsp90)* expression was higher in majority of the treatment groups, including virus- or mock-infected infected bees fed augmented sucrose syrups compared to bees fed non-augmented sucrose syrup except in SINV-infected bees fed thyme oil or 75 ppm fumagillin, which had similar *hsp90* expression levels to bees fed sucrose only. **(C)** *Heat shock 70-kDa protein cognate 4 (hsc70-4)* expression was higher in the majority of treatment groups including mock and virus-infected bees fed sucrose syrup containing either thyme oil (0.16 ppb), fumagillin (25 ppm or 75 ppm) or clothianidin (1 ppb or 10 ppb), except in SINV-infected bees fed sucrose syrup containing thyme oil, which had *hsc70-4* expression levels similar to the controls. Data were analyzed by a pairwise Wilcoxon Rank Sums with a Benjamini–Hochberg correction for multiple comparisons. Asterisks indicate a significant change in gene expression compared to sucrose only control; significance levels: *p < 0.05; **p < 0.005; ***p < 0.0005. This figure shows representative biological replicate for *pl2* expression (i.e., rep1). The data for all three biological replicates for *pl2* are presented in **Supplemental Figure S6** and raw data are included in **Supplemental Tables S11–S16**.

The heat shock response may be induced by a variety of stressors; therefore, we examined the expression of a subset of heat shock response genes (i.e., *pl2*, *hsp90*, and *hsc70-4*) in the context of virus infection and/or chemical exposure. In general, the expression of heat shock protein encoding genes was higher in virus and mock-infected bees fed sucrose syrup containing thyme oil (0.16 ppb), fumagillin (25 ppm or 75 ppm) or clothianidin (1 ppb or 10 ppb), indicating that the expression of these genes is also induced in response to chemical stressors (**Figure 4** and **Supplemental Figure S6**).

In this study, *pl2* exhibited the highest induction in response to virus-infection (i.e., FHV, DWV, and SINV) and chemical stress (**Figure 4A**). In FHV-infected bees, *pl2* expression in bees fed thyme oil augmented sucrose syrup was similar to bees fed non-augmented sucrose syrup, but *pl2* expression was greater in bees that ingested sucrose syrup containing 25 ppm fumagillin (1.42 fold, *p* < 0.001), 75 ppm fumagillin (2.26 fold, *p* < 0.001), 1 ppb clothianidin (2.69 fold, *p* < 0.001), and 10 ppb clothianidin (1.55 fold, *p* < 0.01) compared to FHV-infected bees fed only sucrose syrup. Similarly, DWV-infected bees exhibited higher *pl2* expression when fed sucrose syrup containing 0.16 ppb thyme oil (3.32 fold, *p* < 0.001), 25 ppm fumagillin (2.7 fold, *p* < 0.001), 75 ppm fumagillin (2.65 fold, *p* < 0.001), 1 ppb clothianidin (1.69 fold, *p* < 0.001), and 10 ppb clothianidin (3.74 fold, *p* < 0.001) relative to DWV-infected bees fed sucrose syrup only (**Figure 4A**). In SINV-infected bees, *pl2* expression was higher in bees fed sucrose syrup containing thyme oil (2.3 fold, *p* < 0.01), 25 ppm fumagillin (3.63 fold, *p* < 0.01), 75 ppm fumagillin (4.57 fold, *p* < 0.01), 1 ppb clothianidin (3.97 fold, *p* = 0.023), and 10 ppb clothianidin (3.58 fold, *p* < 0.01) compared to bees fed sucrose syrup only (**Figure 4A**). The expression level of *pl2* was higher in mock-infected bees fed sucrose syrup containing 25 ppm fumagillin (1.27 fold, *p* < 0.001), 75 ppm fumagillin (1.35 fold, *p* < 0.01), and 1 ppb clothianidin (2.04 fold, *p* < 0.001) relative to sucrose only fed bees (**Figure 4A**). Whereas *pl2* expression in mock-infected bees fed sucrose syrup containing thyme oil or 10 ppb clothianidin was similar to levels in mock-infected bees fed only sucrose syrup (*p* > 0.05). In two additional biological replicates, *pl2* expression was greater in virus or mock-infected bees fed sucrose syrup containing thyme oil (0.16 ppb), fumagillin (25 ppm or 75 ppm) or clothianidin (1 ppb or 10 ppb), except in a few treatment groups that exhibited *pl2* levels similar to those observed in honey bees fed sucrose syrup alone [i.e., in rep2, DWV-infected bees fed 25 ppm fumagillin; in rep3, FHV-infected bees fed 0.16 ppb thyme oil augmented sucrose syrup and DWV-infected bees fed 75 ppm fumagillin containing sucrose syrup (**Supplemental Figure S6**)].

The expression of *heat shock protein 90 (hsp90)*, which encodes a protein chaperone that is involved in RISC assembly and siRNA loading, was greater in several treatment groups (169, 170) (**Figure 4**). Specifically, *hsp90* expression was higher in FHV-infected bees fed sucrose syrup containing thyme oil (1.26 fold, *p* = 0.037), 25 ppm fumagillin (1.36 fold, *p* < 0.001), 75 ppm fumagillin (1.54 fold, *p* < 0.001), 1 ppb clothianidin (1.5 fold, *p* = 0.004), and 10 ppb clothianidin (1.26 fold, *p* = 0.003) compared to expression levels in bees fed only sucrose syrup (**Figure 4B**). Likewise, the expression of *hsp90* in DWV-infected bees was higher in bees fed sucrose syrup containing thyme oil (1.38 fold, *p* < 0.001), 25 ppm fumagillin (2.02 fold, *p* < 0.001), 75 ppm fumagillin (1.49 fold, *p* < 0.001), 1 ppb clothianidin (1.63 fold, *p* < 0.001), and 10 ppb clothianidin (1.33 fold, *p* < 0.001), relative to sucrose syrup fed bees (**Figure 4B**). While in SINV-infected bees fed sucrose syrup augmented with 0.16 ppb thyme oil, *hsp90* expression was similar to bees fed only sucrose syrup. The expression of *hsp90* was higher in SINV-infected bees fed sucrose syrup containing 25 ppm fumagillin (2.15 fold, *p* = 0.001), 75 ppm fumagillin (1.44 fold, *p* = 0.017), 1 ppb clothianidin (2.24 fold, *p* = 0.001), and 10 ppb clothianidin (1.81 fold, *p* = 0.001), relative to sucrose syrup fed bees (**Figure 4B**). Expression of *hsp90* was also greater in mock-infected bees fed sucrose syrup containing thyme oil (1.45 fold, *p* < 0.001), 25 ppm fumagillin (1.2 fold, *p* = 0.001), 75 ppm fumagillin (1.31 fold, *p* = 0.017), 1 ppb clothianidin (1.48 fold, *p* < 0.001), and 10 ppb clothianidin (1.14 fold, *p* < 0.001), compared to sucrose syrup fed bees (**Figure 4B**).

*Heat shock protein 70 kDa cognate 4 (hsc70-4)* is a constitutively expressed molecular chaperone and functions with the cooperation of co-chaperones. It is involved in RISC assembly and maintenance of cellular protein homeostasis, including protein folding, assembly, disassembly, and degradation (171–173). Expression of *hsc70-4* was higher in FHV-infected bees fed sucrose syrup containing thyme oil (1.12 fold, *p* < 0.001), 25 ppm fumagillin (1.23 fold, *p* < 0.001), 75 ppm fumagillin (1.79 fold, *p* < 0.001), 1 ppb clothianidin (2.01 fold, *p* < 0.001), and 10 ppb clothianidin (1.32 fold, *p* = 0.016) relative to sucrose syrup fed bees (**Figure 4C**). DWV-infected bees fed sucrose syrup containing either stimulant (thyme oil) or stressors (fumagillin or clothianidin) exhibited greater *hsc70-4* expression than bees fed sucrose syrup only i.e., thyme oil- 1.42 fold; 25 ppm fumagillin- 2 fold; 75 ppm fumagillin- 1.69 fold; 1 ppb clothianidin- 1.65 fold; and 10 ppb clothianidin-1.21 fold (*p* < 0.001) (**Figure 4C**). While SINV-infected bees fed thyme oil augmented sucrose syrup had similar *hsc70-4* expression relative to SINV-infected bees fed only sucrose syrup, SINV-infected bees fed chemical stressors exhibited greater *hsc70-4* expression i.e., 25 ppm fumagillin (2.07 fold, *p* = 0.001), 75 ppm fumagillin (1.4 fold, *p* = 0.024), 1 ppb clothianidin (2.1 fold, *p* = 0.001), and 10 ppb clothianidin (1.79 fold, *p* = 0.001) (**Figure 4C**). *Hsc70-4* expression was greater in mock-infected honey bees fed either augmented diets, compared to those fed only sucrose syrup i.e., thyme oil- 1.76 fold; 25 ppm fumagillin- 1.5 fold; 75 ppm fumagillin- 1.39 fold; 1 ppb clothianidin- 1.94 fold; and 10 ppb clothianidin- 1.87 fold relative to sucrose syrup fed bees (*p* < 0.001) (**Figure 4C**). Since analyses of *hsp90* and *hsc70-4* expression in one biological replicate was similar to *pl2* expression data indicating that in general honey bee heat shock protein encoding genes exhibited higher expression in response to all chemicals, including thyme oil, fumagillin, and clothianidin, additional biological replicates were not analyzed for *hsp90* and *hsc70-4* expression.

#### 2.2.3 Greater Expression of Antimicrobial Peptide Encoding Genes in Thyme Oil Fed Bees, Reduced in Fumagillin and Clothianidin Fed Bees

**Figure 5:**
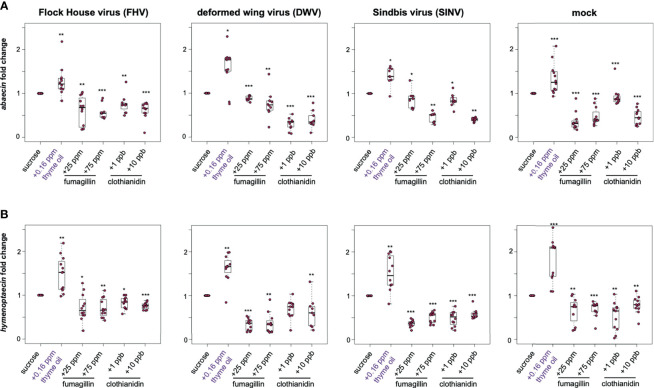
Expression of antimicrobial peptides encoding genes was greater in bees fed thyme oil augmented sucrose syrup and lower in bees fed fumagillin or clothianidin containing sucrose syrup. The expression of two antimicrobial peptides (*abaecin* and *hymenoptaecin*) was assessed in mock and virus-infected bees fed sucrose syrup containing additives. **(A)** In virus- and mock-infected bees (i.e., FHV, DWV, SINV), *abaecin* expression was higher in bees fed sucrose syrup containing thyme oil and lower in bees fed sucrose syrup containing fumagillin (25 ppm or 75 ppm) or clothianidin (1 ppb or 10 ppb) compared to bees fed sucrose only. **(B)** In virus-infected bees (i.e., FHV, DWV, SINV), *hymenoptaecin* expression was higher in bees fed sucrose syrup containing thyme oil and lower in bees fed sucrose syrup containing fumagillin (25 ppm or 75 ppm) or clothianidin (1 ppb or 10 ppb). *Hymenoptaecin* expression was higher in mock-infected bees fed sucrose syrup augmented with 0.16 ppb thyme oil, but lower in mock-infected bees fed either fumagillin (i.e., 25 ppm or 75 ppm) or clothianidin (1 ppb or 10 ppb) containing sucrose syrup. Data were analyzed by a pairwise Wilcoxon Rank Sums with a Benjamini–Hochberg correction for multiple comparisons. Asterisks indicate a significant change in gene expression compared to sucrose only control; significance levels: *p < 0.05; **p < 0.005; ***p < 0.0005. This figure shows representative biological replicate for the expression of each gene (i.e., rep1 for *abaecin* and rep3 for *hymenoptaecin*). The data for one additional biological replicate is presented in **Supplemental Figures S7**, **S8**. Raw data are included in **Supplemental Tables S17–S20**.

The honey bee genome encodes a suite of antimicrobial proteins (AMPs) that are expressed in response to microbial pathogens. Although the role of these genes in the context of virus infections in insects has not been well-characterized, increased expression is commonly used as a hallmark of active immune signaling pathways including the Toll, Imd, and Jak/STAT pathways [reviewed in (37, 40, 41)]. Therefore, we examined the expression of two honey bee AMP encoding genes *abaecin* and *hymenoptaecin* as an indicator of immune pathway status in virus-infected bees fed chemically augmented sucrose syrup. *Abaecin* is an effector molecule expressed as a result of an immune challenge that triggers the induction of Toll and Imd pathways (174–176). *Abaecin* expression in FHV-infected honey bees fed sucrose syrup supplemented with thyme oil was 1.28 fold higher relative to those fed only sucrose syrup (*p* = 0.001) (**Figure 5A**). In contrast, *abaecin* expression in FHV-infected bees fed either fumagillin or clothianidin was slightly lower than expression in FHV-infected bees fed plain sucrose syrup (i.e., 25 ppm fumagillin, 0.61 fold, *p* = 0.001; 75 ppm fumagillin, 0.59 fold, *p* < 0.001; 1 ppb clothianidin, 0.75 fold, *p* = 0.002; and 10 ppb clothianidin, 0.63 fold, *p* < 0.001) (**Figure 5A**). DWV-infected bees fed thyme oil augmented sucrose syrup had 1.58 fold greater *abaecin* expression than sucrose syrup fed bees (*p* = 0.029). Whereas chemically stressed DWV-infected bees exhibited reduced *abaecin* expression than DWV-infected bees fed sucrose syrup alone (i.e., 25 ppm fumagillin, 0.88 fold, *p* < 0.001; 75 ppm fumagillin, 0.76 fold, *p* = 0.003; 1 ppb clothianidin, 0.3 fold, *p* < 0.001; and 10 ppb clothianidin, 0.4 fold, *p* < 0.001) (**Figure 5A**). Likewise, SINV-infected honey bees fed thyme oil augmented sucrose syrup had 1.4 fold higher *abaecin* expression compared to sucrose syrup fed bees (*p* = 0.01). Whereas SINV-infected bees fed chemical stressors exhibited reduced *abaecin* expression (i.e., 25 ppm fumagillin, 0.87 fold, *p* = 0.01; 75 ppm fumagillin, 0.45 fold, *p* = 0.001; 1 ppb clothianidin, 0.85 fold, *p* = 0.01; and 10 ppb clothianidin, 0.41 fold, *p* = 0.001) (**Figure 5A**). The same trend was also observed in mock-infected bees, which exhibited 1.33 fold higher *abaecin* expression in bees fed thyme oil augmented sucrose syrup compared to levels in bees fed non-augmented sucrose syrup (*p* < 0.001). Whereas *abaecin* expression was reduced in mock-infected bees fed sucrose syrup containing 25 ppm fumagillin (0.36 fold, *p* < 0.001), 75 ppm fumagillin (0.47 fold, *p* < 0.001), 1 ppb clothianidin (0.92 fold, *p* < 0.001), and 10 ppb clothianidin (0.47 fold, *p* < 0.001) (**Figure 5A**). In general, similar trends in *abaecin* expression was observed in one additional biological replicate except *abaecin* expression in FHV-infected bees fed sucrose syrup containing 75 ppm fumagillin or DWV-infected bees fed 1 ppb clothianidin was similar to expression levels in bees fed sucrose syrup (**Supplemental Figure S7**, *p* > 0.05).

*Hymenoptaecin* is an antimicrobial peptide encoding gene that is induced *via* the nuclear factor kappa B (NF-κB) signaling in Imd pathway (175). In the previous study by Palmer-Young et al., expression of *hymenoptaecin* in laboratory contained bees fed 0.16 ppb thymol for 7 days exhibited 12.9 to 61-fold greater expression than control bees (104). In our study, the overall trend in *hymenoptaecin* expression in bees that were either mock- or virus-infected and fed sucrose syrup containing thyme oil was higher, whereas in bees fed fumagillin, or clothianidin were lower relative to bees fed sucrose syrup (**Figure 5B**). FHV-infected bees fed thyme oil containing sucrose syrup had 1.48 fold higher expression of *hymenoptaecin* compared to bees fed sucrose syrup (*p* = 0.002). FHV-infection coupled with fumagillin or clothianidin containing sucrose syrup resulted in lower levels of *hymenoptaecin* expression compared to levels in bees fed plain sucrose syrup (i.e., 25 ppm fumagillin, 0.72 fold, *p* = 0.019; 75 ppm fumagillin, 0.7 fold, *p* = 0.002; 1 ppb clothianidin, 0.83 fold, *p* = 0.027; and 10 ppb clothianidin, 0.76 fold, *p* < 0.001) (**Figure 5B**). Likewise, DWV-infected honey bees fed thyme oil augmented sucrose syrup had 1.62 fold higher *hymenoptaecin* expression relative to bees fed sucrose syrup only (*p* = 0.002). However, *hymenoptaecin* expression was lower in DWV-infected bees fed sucrose syrup containing 25 ppm fumagillin (0.34 fold, *p* < 0.001), 75 ppm fumagillin (0.36 fold, *p* < 0.001), 1 ppb clothianidin (0.69 fold, *p* = 0.001), and 10 ppb clothianidin (0.62 fold, *p* = 0.006) than levels in bees fed only sucrose syrup (**Figure 5B**). SINV-infected bees fed sucrose syrup supplemented with 0.16 ppb thyme oil exhibited 1.51 fold greater expression of *hymenoptaecin*, relative to bees fed sucrose syrup only (*p* = 0.001). Bees infected with SINV and fed fumagillin or clothianidin containing sucrose syrup had lower levels of *hymenoptaecin* expression compared to bees fed sucrose syrup alone (i.e., 25 ppm fumagillin, 0.37 fold; 75 ppm fumagillin, 0.51 fold; 1 ppb clothianidin, 0.48 fold; and 10 ppb clothianidin, 0.58 fold; all with *p* < 0.001) (**Figure 5B**). Similar trends in *hymneoptaecin* expression were observed in mock-infected bees fed thyme oil, which exhibited 1.8 fold greater expression relative to bees fed sucrose syrup (*p* < 0.001), whereas mock-infected bees exposed to chemical stressors had reduced *hymenoptaecin* expression levels (i.e., in 25 ppm fumagillin, 0.66 fold, *p* = 0.004; 75 ppm fumagillin, 0.68 fold, *p* < 0.001; 1 ppb clothianidin, 0.52 fold, *p* = 0.004; and 10 ppb clothianidin, 0.78 fold, *p* = 0.004) (**Figure 5B**). Similar trends in *hymenoptaecin* expression were observed in one additional biological replicate of this experiment except in rep1, *hymenoptaecin* expression in bees fed thyme oil and either infected with FHV or SINV exhibited similar levels of expression to bees fed only sucrose syrup (**Supplemental Figure S8**).

#### 2.2.4 Greater *Vitellogenin* Expression in Thyme Oil Fed Bees, Reduced in Fumagillin and Clothianidin Fed Bees

**Figure 6:**
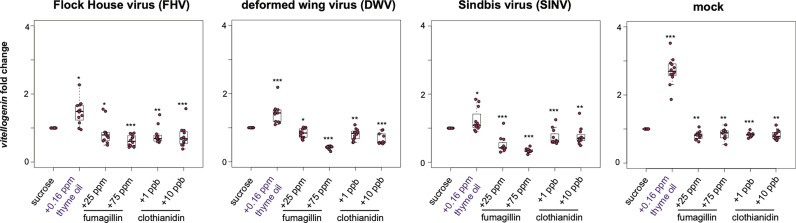
*Vitellogenin* expression was higher in bees fed sucrose syrup containing thyme oil and lower in bees fed sucrose syrup containing fumagillin or clothianidin. *Vitellogenin* expression was assessed using qPCR and the relative gene expression was analyzed using the ΔΔCt method with normalization to *rpl8* and relative to expression in mock or virus-infected bees fed sucrose syrup only. In virus-infected (i.e., FHV, DWV, SINV) and mock-infected honey bees *vitellogenin* expression was greater in bees fed sucrose syrup containing thyme oil and reduced in bees fed sucrose syrup containing fumagillin (25 ppm or 75 ppm) or clothianidin (1 ppb or 10 ppb) relative to sucrose only fed bees. Asterisks indicate a significant change in gene expression compared to sucrose only control; significance levels: *p < 0.05; **p < 0.005; ***p < 0.0005. This figure shows representative biological replicate for *vitellogenin* expression. The data for one additional biological replicate is presented in **Supplemental Figure S9**. Raw data are included in **Supplemental Tables S21**, **S22**.

In honey bees, *vitellogenin* (*vg*) has numerous functions including roles in immunity and stress resistance [reviewed in (77)]. Overall, the trends in *vg* expression were similar to the trends observed for other honey bee immune genes including *dcr-like*, *ago2*, *abaecin*, and *hymeptaecin*. Virus infection in conjugation with thyme oil augmented diets resulted in higher levels of *vg* expression compared to virus-infected bees fed sucrose only (i.e., FHV- 1.47 fold, *p* = 0.023; DWV- 1.42 fold, *p* < 0.001; SINV- 1.23 fold, *p* = 0.01). The expression of *vg* was highest for 0.16 ppb thyme oil fed, mock-infected bees, which exhibited 2.71 fold greater expression than bees fed sucrose only (*p* = 0.0001). Whereas virus-infected bees fed either fumagillin or clothianidin containing diets had lower expression of *vg*. Specifically, in FHV-infected bees fed chemical containing sucrose syrup resulted in reduced *vg* expression (i.e., 25 ppm fumagillin, 0.86 fold, *p* = 0.023; 75 ppm fumagillin, 0.62 fold, *p* = 0.002; 1 ppb clothianidin, 0.77 fold, *p* < 0.001; and 10 ppb clothianidin, 0.74 fold, *p* = 0.002) compared to bees fed sucrose syrup (**Figure 6**). Analogously, dual stressed bees resulted in reduced *vg* expression in DWV-infected bees fed sucrose syrup containing 25 ppm fumagillin (0.84 fold, *p* = 0.013), 75 ppm fumagillin (0.42 fold, *p* < 0.001), 1 ppb clothianidin (0.8 fold, *p* = 0.001), and 10 ppb clothianidin (0.68 fold, *p* < 0.001) compared to bees fed sucrose syrup (**Figure 6**). *Vitellogenin* expression was reduced in SINV-infected bees fed with sucrose syrup containing 25 ppm fumagillin (0.51 fold, *p* < 0.001), 75 ppm fumagillin (0.34 fold, *p* < 0.001), 1 ppb clothianidin (0.73 fold, *p* < 0.0001), and 10 ppb clothianidin (0.77 fold, *p* = 0.002) compared to bees fed sucrose syrup (**Figure 6**). In general, similar trends in *vg* expression were observed in mock-infected bees exposed to a single stressor, i.e., in bees fed sucrose syrup containing 25 ppm fumagillin (0.81 fold, *p* = 0.001), 75 ppm fumagillin (0.85 fold, *p* = 0.001), 1 ppb clothianidin (0.83 fold, *p* < 0.001), and 10 ppb clothianidin (0.82 fold, *p* = 0.001) compared to bees fed sucrose syrup (**Figure 6**). The expression of *vg* as analyzed in one additional biological replicate followed similar trends except in rep1, FHV-infected bees fed 10 ppb clothianidin and SINV-infected bees fed 25 ppm fumagillin, *vitellogenin* expression was constant relative to bees fed sucrose syrup (**Supplemental Figure S9**).

## 3 Discussion

Honey bee health is impacted by numerous factors including pathogens and chemical stressors. These stressors impact honey bee health at the colony, individual bee, and cellular levels. Honey bees have evolved various strategies to mitigate these threats (97, 177–179). However, the impact of multiple, simultaneous biotic and abiotic stressors on honey bee health and longevity is currently not well understood, and difficult to assess at the colony level due to the large number of confounding variables (e.g., weather, colony management, pathogen exposure, co-infections). Therefore, we performed laboratory-based experiments to examine the impact of virus infection in the context of putative chemical stimulant (i.e., thyme oil) and stressors (i.e., fumagillin and clothianidin), while limiting the effects of additional compounding variables. The results described herein demonstrate that honey bees fed sucrose syrup supplemented with 0.16 ppb thyme oil harbored 25-87% less virus than bees fed only sucrose syrup. This trend was consistent for a panel of viruses including FHV, SINV-GFP, and DWV. Honey bees fed thyme oil exhibited greater expression of genes involved in antiviral defense including the RNAi pathway (i.e., *ago2* and *dcr-like*) and antimicrobial peptide genes regulated by Toll and Imd pathways (i.e., *abaecin* and *hymenoptaecin*), as well as a gene involved in immune competence and a marker of the overall bee health, *vitellogenin.* Together these results indicate that thyme oil stimulates honey bee immune responses and mitigates virus infections.

This is in line with previous studies that have documented modulation of honey bee gene expression in response to phytochemicals. Specifically, in the context of natural DWV infections, Palmer-Young et al. demonstrated greater *hymenoptaecin* expression (12.9 to 61-fold) in honey bees fed 0.16 ppb thymol relative to controls (104). Likewise, Mao et al. demonstrated that honey bees fed *p*-coumaric acid, a common pollen phytochemical, had greater expression of two other antimicrobial peptide encoding genes (i.e., 1.2 fold *defensin* and 1.9 fold *abaecin*), as well as genes involved in detoxification (107). Our results, coupled with previous studies are promising and may indicate that thyme oil augmented diets, either naturally acquired or fed could be used to enhance individual bee immunocompetence and, in turn, mitigate virus-associated colony deaths. However, further investigation is required to determine the range of thymol concentrations that positively impact honey bee health, since exposure to higher doses of thymol (i.e., 16 ppm) were lethal in previous studies (104). Additional studies are also required to further investigate the precise mechanisms of action, while results to date suggest thymol stimulates the expression of key honey bee immune genes. The signal transduction cascades, sensors, transcription factors, and effector proteins involved have yet to be fully elucidated.

The impact of agrochemicals, including neonicotinoid insecticides on honey bee health and colony longevity is an important area of research that includes a large body of literature including numerous studies that have examined field relevant exposure, colony longevity, and the impacts on individual bees in laboratory-based experiments (139, 180–182). While acute toxicity is easy to measure in laboratory-based studies, sublethal impacts are more difficult to assess. Furthermore, the impact of sublethal doses of chemicals, including clothianidin, on the immune response and in turn the outcome of virus infection is even more difficult to discern. Previous studies demonstrated a negative impact of clothianidin (10 ppb) on the activation of Toll (NF-κB/Dorsal) pathway in honey bee larvae (154, 183). Clothianidin exposure enhances the transcription of the NF-κB inhibitor and suppresses the immune response. This eventually results in higher DWV replication (154). Similar to previous results, we determined that honey bees fed sucrose containing clothianidin (1 ppb) had reduced expression of key immune genes (i.e., *ago2, dcr-like, abaecin, hymenoptaecin*, and *vitellogenin*), which resulted in more severe virus infections, using virus abundance as a proxy for infection severity for a panel of viruses. Together our results coupled with those from previous studies, suggest that sublethal doses of clothianidin may increase the overall virus burden in a colony. However, honey bees fed sucrose syrup containing 10 ppb clothianidin had virus levels that were similar to, or lower than, levels in bees fed only sucrose syrup. Although additional studies are needed, we hypothesize that bees fed 10 ppb clothianidin may have been less suitable hosts for viruses, which are obligate intracellular pathogens and, thus, rely on healthy host cells for replication. An alternative hypothesis, which is not well supported by the data described herein is that reduced virus abundance in 10 ppb clothianidin fed bees is due to hormesis (i.e., a dose-dependent beneficial biological response to a chemical stressor) (184, 185). Insecticide-induced hormesis has been reported to have ‘positive’ effects on survival, reproduction, and immunity in bees and other insects and is involved in various life stages and different insecticide active ingredients (184–187). The possibility of virus reduction due to hormetic effects was mentioned in another study that found DWV-infected honey bees fed 0.12 - 0.35 ng clothianidin per bee (~ 1.2 - 3.5 ppb) had lower DWV levels than bees fed 0.05 ng clothianidin per bee (~ 0.5 ppb) (143). However, in our study, the beneficial virus limiting response in bees fed 10 ppb clothianidin did not correspond with greater immune gene expression, therefore the hormetic effect hypothesis is not well-supported.

Beekeepers use in-hive chemical treatments as part of integrated pest management (IPM) strategies to maintain health honey bee colonies. These beekeeper-applied treatments include miticides and the antifungal compound fumagillin, which is used to mitigate *Nosema* spp. infections. Previous studies indicated that fumagillin treatments may negatively impact bee health, therefore a limit of two treatments per colony is included as a label advisory. To further investigate the impacts of fumagillin treatment on the honey bee immune system and the outcome of virus infection, honey bees were fed sucrose containing the recommended dose of 25 ppm, or a greater dose of 75 ppm. We determined that virus abundance was higher in bees fed sucrose syrup containing 25 ppm or 75 ppm fumagillin, relative to bees fed sucrose syrup only. The greater virus abundance is likely due to immune suppression, as the expression of select honey bee immune genes (i.e., *ago2, dcr-like, abaecin, hymenoptaecin*, and *vitellogenin*) were reduced in fumagillin fed bees. Although future studies are warranted before adjusting recommendations for honey bee colony management, our results suggest that beekeepers should consider trade-off of reducing *Nosema* infections, but increasing virus abundance. This consideration may be more important at the end of the beekeeping season in temperate climates (i.e., late autumn), since DWV infections are usually greater at that time of year (15, 27, 28). While the focus of our experiments was to evaluate the impact of dual stressors on individual honey bee health, we determined that honey bees exposed to a single stressor, i.e., fumagillin (25 ppm or 75 ppm) or clothianidin (1 ppb) had reduced levels of key immune genes (i.e., *ago2, dcr-like, abaecin, hymenoptaecin*, and *vitellogenin)* compared to bees fed sucrose syrup alone. The reduced expression of *vitellogenin* in fumagillin or clothianidin (1 ppb) fed bees indicates poor health status of the host while the reduced expression of key immune genes suggests hindered immune responses.

Collectively, the results described herein indicate that 0.16 ppb thyme oil acts as an immunostimulant in honey bees and results in reduced levels of virus infection. Additional studies that further examine the kinetics of immune stimulation and the impact of multiple treatments on virus infections over a longer time frame are required to help move these studies beyond the laboratory and towards colony level investigations that may benefit beekeeping operations. It will also be important to examine the impact of simultaneous exposure to putative immune system stimulants including thyme oil, in the context of varying doses of chemical stressors (fungicide and/or insecticide) on the outcome of honey bee virus infections. In addition, chemical adjuvants can negatively impact the outcome of virus infections in the context of other chemical stressors, and therefore their impact also requires further investigation (188). Studies that more clearly mimic the real-world multifactorial and variable biotic and abiotic stressors encountered by honey bees, and other beneficial insect pollinators, will be instrumental to the development of strategies aimed at mitigating their losses (11, 79, 133, 189–192).

## 4 Materials and Methods

### 4.1 Honey Bees

Honey bee colonies were originally established as packages containing naturally mated *Apis mellifera carnica* queens and maintained in Langstroth hives in an apiary located at Montana State University’s Horticulture Farm in Bozeman, MT, USA. Frames of capped brood with emerging bees were obtained from established colonies one day prior to each experiment and kept at 32°C and in a laboratory incubator overnight. Age-matched (~24 h post-emergence) adult female, worker bees were collected and housed in modified deli-containers and randomly assigned into treatment groups. Honey bees in each treatment group were maintained in laboratory incubators at 32°C and ~ 20% relative humidity and fed 50% sucrose solution *ad libitum* with or without additives including thyme oil, fumagillin, and clothianidin as described below. Experiments described herein were carried out using honey bees collected from three separate colonies obtained during May-August 2020, designated as rep1, rep2, and rep3.

### 4.2 Virus Preparation

#### 4.2.1 Flock House Virus

Flock House virus (FHV) was propagated in *Drosophila melanogaster* Schneider 2 (S2) cells derived from *D. melanogaster* embryos. S2 cells were grown as monolayers in Schneider’s *Drosophila* medium supplemented with 10% heat-inactivated fetal bovine serum (Life Technologies) and 1% penicillin-streptomycin as per manufacturer’s instructions. To generate FHV particles, 4 x 10^7^ S2 cells/ml were seeded in a sterile T75 flask (Thermo Scientific). An aliquot of FHV was kindly provided by Dr. Anette Schneemann (The Scripps Research Institute, La Jolla, California). S2 cells were infected with FHV at a multiplicity of infection of 1 pfu/cell and incubated at 28°C for 48 h as previously described (193). After incubation, the cells were lysed by addition of 10% (vol/vol) Nonidet P-40 and incubated on ice for 10 min with periodic swirling. Cell debris was pelleted in a Sorvall LYNX 4000 Centrifuge (Thermo Fisher) at 13,800x *g* for 10 min at 4°C and the clarified supernatant was transferred to a fresh tube. Virus was pelleted through a 1 mL volume of 30% (wt/wt) sucrose in 50 mM HEPES (pH 7) at 40,000 rpm for 2.5 h at 11°C in a SW41 Ti Swinging-Bucket Rotor (Beckman Coulter). The pellets were resuspended in 0.5 mL 50 mM HEPES (pH 7) and centrifuged at 10,000 rpm for 10 min to remove any insoluble material. The clarified supernatant was layered on continuous sucrose gradients (40%, 35%, 30%, 25%, 20%, 15% and 10% (wt/wt)) and centrifuged at 40,000 rpm for 1.5 h at 11°C to sediment the virus halfway down the tube. The sedimented virus was further pelleted in 10 mM Tris HCl buffer (pH 7.5) at 40,000 rpm for 2.5 h at 11°C for injections in honey bees. RNA was isolated from 100 µL virus preparations (as described below) and FHV abundance was quantified by RT-PCR and copy number was based on a standard curve. The dose utilized for laboratory-based honey bee infection studies was 3.5 x 10^8^ FHV RNA copies per bee. FHV RNA 2 plasmid standards were used as templates, with concentrations ranging from 10^3^ to 10^9^ copies per reaction to create a linear standard curve, with a limit of detection of 10^3^ copies of FHV cDNA using primers FHV-FW and FHV-REV (**Supplemental Table S1**). The linear equation for the plasmid standard for FHV was Ct = -3.240x + 40.138 (R^2^ = 0.980, efficiency = 103%) where ‘x’ is the log (FHV RNA copies).

#### 4.2.2 Deformed Wing Virus

DWV was propagated in white eyed pupae collected from a frame of capped brood. Specifically, honey bee pupae were injected with 3.41 x 10^7^ DWV RNA copies in 2 μL between the 2^nd^ and 3^rd^ integuments of the abdomen using microcapillary glass needle and a Harbo syringe (Honey bee Insemination Service). Post-injection, pupae were held at 30°C in a humid incubator for the course of infection. Infection progression was tracked by the eye color to ensure the pupae were alive. Live pupae were harvested at 10 days post infection and place in a 2 mL safe-lock Eppendorf tube with 1 mL PBS (pH 7.4) and homogenized using a Tissue Lyser II (Qiagen) at 30 Hz for 2 min. The homogenate was centrifuged at 14,000x *g* for 15 min at 4°C and the clarified supernatant was transferred to a fresh tube. DWV RNA copies were quantified using qPCR to be 3.41 x10^7^ DWV RNA copies/µL. The inoculum was tested for copurifying/contaminating viruses (ABPV, BQCV, CBPV, IAPV, KBV, LSVs, and SBV) *via* PCR. No other viruses except DWV were detected *via* PCR. (**Supplemental Figure S11**). The dose utilized for laboratory-based honey bee infection studies was 3.5 x 10^8^ DWV RNA copies per bee. DWV plasmid standards were used as templates, with concentrations ranging from 10^3^ to 10^9^ copies per reaction to create a linear standard curve, with a limit of detection of 10^3^ copies of DWV cDNA using primers DWV-F 1170 and DWV-R 1364 (**Supplemental Table S1**). The linear equation for the plasmid standard for DWV was Ct = -3.469x + 40.803 (R^2^ = 0.999, efficiency = 94.5%) where ‘x’ is the log (DWV RNA copies).

#### 4.2.3 Sindbis-Green Fluorescent Protein Tagged

We utilized a recombinant model virus, Sindbis virus expressing green fluorescent protein (42, 93, 194). We and others have used SINV to investigate honey bee, fruit fly, and mosquito antiviral defense mechanisms (42, 43, 61, 93). SINV was propagated in Baby Hamster Kidney fibroblast (BHK-21) cells. BHK-21 cells were grown as a monolayer in Eagle’s Minimum Essential Medium with 10% fetal bovine serum. A 90% confluent flask was infected with a multiplicity of infection of 0.001 as previously described (94). Infected cells were incubated at 37°C with 5% carbon dioxide for 24 h. Cells were collected, and the cell debris was removed by centrifugation at 1000x *g* for 10 min. The clarified supernatant was quantified using a standard plaque assay (93). Each bee was injected with 3,750 plaque forming units (PFUs) SINV. SINV-GFP plasmid standards were used as templates, with concentrations ranging from 10^3^ to 10^9^ copies per reaction to create a linear standard curve, with a limit of detection of 10^3^ copies of SINV-GFP cDNA using primers qSindbisFW4495 and qSindbisREV4635 (**Supplemental Table S1**). The linear equation for the plasmid standard for SINV-GFP was Ct = -3.335x + 40.137 (R^2^ = 0.993, efficiency = 99.5%) where ‘x’ is the log (SINV-GFP RNA copies).

### 4.3 Honey Bee Virus Infection

Age-matched (~24 h post-emergence) adult female, worker bees were cold anesthetized at 4°C for 10 min prior to intrathoracic injection of virus or buffer (mock-infection). Intra-thoracic injections were performed using a Harbo syringe (Honey Bee Insemination Service) and microcapillary glass needles, made by pulling borosilicate glass capillary tubes (100 mm long, 1 mL capacity, Kimble-Chase) with a coil temperature of 61°C on the PC-10 Dual-Stage Glass Micropipette Puller (Narishige). Honey bees were infected with 3.5 x 10^8^ FHV RNA copies/bee in 10mM Tris HCl buffer pH 7.5, 3.5 x 10^8^ DWV RNA copies/bee in 10mM Tris HCl buffer pH 7.5 or 3,750 PFUs SINV-GFP. Mock-infected bees were injected with 2 µL buffer (10mM Tris HCl, pH 7.5).

### 4.4 Honey Bee Diet Preparation

Post-injection, honey bees were housed in modified deli containers for the duration of the study. Bees in the control group were fed 50% sucrose syrup only, whereas bees in treatment groups were fed sucrose syrup containing one of the following additives: 0.16 ppb thyme oil (Body wonders), Fumagilin-B^®^, fumagillin dicyclohexyl ammonium (Medivet Pharmaceuticals Ltd.) at manufacturer’s recommended dose of 25 ppm or a higher dose of 75 ppm, or clothianidin at the field relevant sublethal concentration of 1 ppb or near lethal dose of 10 ppb (130, 195, 196). Thyme oil contains 10% - 64% thymol (~37% average) depending on the plant species, geographical sources, and harvest season, which may affect the volatile composition of the plant (113, 116–118). We estimate that 0.16 ppb thyme oil used in this experiment may contain approximately 0.06 ppb (60 ppb) thymol (37% thymol in 0.16 ppb thyme oil corresponds to ~ 60 ppb). For clothianidin treatments we utilized the commercially available Poncho^®^ 600, which contains 48% of the active ingredient clothianidin. A working stock of 1000 ppb clothianidin was prepared by 1:10 serial dilutions in 50% sucrose solution, which was further diluted in 50% sucrose to prepare 1 ppb (i.e., 10 ul of 1000 ppb Poncho^®^ 600) and 10 ppb (i.e., 100 ul of 1000 ppb Poncho^®^ 600) clothianidin solutions. Honey bees were fed sucrose solution either alone or with additives using cage feeders that were made by putting two holes on each side of a 1.5 mL centrifuge tube; sucrose solution was checked daily and refilled as needed throughout the study.

### 4.5 RNA Isolation

Individual honey bee sample was dissected into head, thorax, and abdomen. The abdomen was utilized for further analysis since it is distal to the injection site and thus virus infection naturally spread into that tissue, and it contains the immune cell generating fat body. Honey bee abdomens were transferred into 2 mL Eppendorf safe-lock tubes and homogenized in 300 µL deionized water with a sterile steel ball (5 mm) using a Tissue Lyser II (Qiagen) at 30 Hz for 2 min. An equal volume (300 µL) of TRIzol reagent (Invitrogen) was added to homogenate and then samples were vortexed for 15 s followed by a 5 min incubation at room temperature. Next, 100 µL chloroform was added and each sample was shaken by hand for 15 s and incubated at room temperature for 2 min. Samples were then centrifuged at 12,000x *g* at 4°C for 15 min and the aqueous phase was transferred to a fresh 1.5 mL centrifuge tube. An equal volume of isopropanol was added to the aqueous phase, vortexed, and incubated at room temperature for 10 min to precipitate the nucleic acid. The nucleic acid was pelleted by centrifugation at 12000x *g* at 4°C for 10 min. Supernatant was carefully removed and the pellets were washed with 75% ethanol by centrifugation at 7500x *g* at 4°C for 5 min. The resulting supernatant was discarded, and the pellets were air dried for 10 min at room temperature, then dissolved in 50 µL deionized water. RNA concentrations were assessed on Nanodrop 2000 spectrophotometer (Thermo Fisher). All RNA samples were stored in -80°C until further analysis.

### 4.6 Reverse Transcription/cDNA Synthesis

Reverse transcription reactions were carried out to synthesize complimentary DNA (cDNA) by incubating 2,000 ng of RNA with M-MLV reverse transcriptase (Promega) and 500 ng random hexamer primers (IDT) for 1 h at 37°C according to manufacturer’s instructions. The resulting cDNA was diluted 1:2 with sterile distilled water.

### 4.7 Polymerase Chain Reaction

Polymerase chain reaction (PCR) was used to test mock-infected bees in all experimental replicates for pre-existing infections with honey bee infecting viruses, since bees were obtained from honey bee colonies that may have varying levels of naturally prevalent bee infecting viruses (i.e., BQCV, CBPV, ABPV, DWV, IAPV, KBV, SBV, LSVs 1–4). To reduce the number of individual PCR tests, individual bee cDNA samples (n= 11-12) were pooled, resulting in three pooled samples per treatment. Each pool was tested for pre-existing virus infections using pathogen-specific primers (**Supplemental Table S1** and **Supplemental Figure S10**). PCR was performed according to standard methods (197). In brief, 2 µL cDNA template was combined with 10 pmol of each forward and reverse primer, and amplified with Denville ChoiceTaq polymerase (Thomas Scientific) according to the manufacturer’s instructions using the following cycling conditions: 95°C for 5 min, 95°C for 30 s, 57°C for 30 s, 72°C for 30s, 35 cycles, followed by final elongation at 72°C for 4 min.

### 4.8 Quantitative PCR

Quantitative PCR was used to quantify relative abundances of viruses and honey immune gene transcripts using the primers listed in **Supplemental Table S1**. All qPCR reactions were performed in triplicate with 2 μL of cDNA template. Each 20 μL reaction contained 1× ChoiceTaq Mastermix (Thomas Scientific), 0.4 μM each forward and reverse primer, 1× SYBR Green (Life Technologies), and 3 mM MgCl_2_. A CFX Connect Real Time instrument (BioRad) was used to carry out reactions with the following thermo-profile: pre-incubation at 95°C for 1 min followed by 40 cycles of 95°C for 10 s, 60°C for 20 s, and 72°C for 15 s; final extension at 72 °C for 1 min, followed by melt curve analysis. Reactions without template were carried out as negative controls. The qPCR specificity was verified through melt point analysis and gel electrophoresis of select samples. The sequence amplicons were previously verified by chain-termination sequencing. As described above in section 4.2 for virus stock quantification, virus abundance in individual bee samples was determined relative to a standard curve that was generated by the use of virus amplicon specific plasmids, with concentrations ranging from 10^3^ to 10^9^ copies per reaction, as templates to create linear standard curves. The linear equations for each virus amplicon are included in section 4.2 above. The qPCR and mortality data for individual samples is included in **Supplemental Tables S3**, **S5**. The virus fold change for dead and live virus-infected honey bees fed chemical treatments was plotted for each treatment group and no significant difference in fold change was recorded in dead vs. live bees. Thus, in our analyses, data from dead and live bees was combined and presented.

To assess the relative expression of honey bee genes including immune genes (i.e., *argonaute-2, dicer-like, abaecin, hymenoptaecin, heat shock protein 90, heat shock protein 70 cognate 4, protein lethal (2) essential for life-like*), and *vitellogenin*, relative to the expression of a reference gene (i.e., *ribosomal protein L8*) was carried out using the gene-specific primer sets in **Supplemental Table S1** and the ΔΔCt method (198). Specifically, the relative expression of host genes was determined by a ranked ΔΔCt method in which the ΔCt was calculated by subtracting the rpl8 Ct value from the Ct of the gene of interest. Then, the within-group ΔCt values were ranked from highest to lowest, and the relevant corresponding control ΔCt value was subtracted from the treatment group ΔCt to obtain the ΔΔCt. The fold-change in cDNA abundance was calculated by the equation 2^−ΔΔCt^. Efficiency of each primer set was evaluated using cDNA dilution series and calculated by plotting log_10_ of the concentration versus the crossing point threshold (C(t)) values and using the primer efficiency equation (10^(1/Slope)-1^) x 100). The efficiency equations of the primers utilized for honey bee immune genes are- *ago-2* Cp= -3.36x + 30.28, R² = 0.99; *dcr-like* Cp = -3.56x + 31.22, R² = 0.99; *abaecin* Cp = -3.37x + 29.8, R² = 0.99; *hymenoptaecin* Cp = -3.44x + 30.50, R² = 0.99; *hsp90* Cp = -3.43x + 32.98, R² = 0.99; *hsc70-4* Cp = -3.36x + 31.52, R² = 0.99; *pl2* Cp = -3.16x + 30.12, R² = 0.99; *vitellogenin Cp* = -3.04x + 29.44, R² = 0.99.

### 4.9 Statistical Analysis

All data were analyzed using R v4.0.2 in RStudio V1.3.1073. Pairwise comparisons for virus abundance and gene expression were evaluated using the *pairwise.wilcox.test* function in the base R stats package to perform a Wilcoxon Rank Sums test with Benjamini-Hochberg correlation for multiple comparisons (199).

## Data Availability Statement

The majority of the data generated or analyzed during this study are included in this published article and its **Supplementary Material** files (available online). Additional data are available from the corresponding author upon request.

## Author Contributions

Conceptualization, FP, KD, and MF. Methodology, FP, KD, and MF. Formal analysis, FP. Investigation, FP and MF. Resources, MF. Data curation, FP and MF. Writing—original draft preparation, FP and MF. Writing—review and editing, FP and MF. Visualization, FP and MF. Supervision, MF. Project administration, MF. Funding acquisition, MF. All authors have read and agreed to the published version of the manuscript.

## Funding

The Flenniken laboratory is supported by the National Science Foundation CAREER Program (Award Number 1651561), Montana Department of Agriculture Specialty Crop Block Grant Program (Award Number 19SCG04703), and United States Department of Agriculture National and Food Research Initiative (USDA-NIFA) Hatch Multistate Funding (NC-1173). The funders had no role in study design, data collection and analysis, decision to publish, or preparation of this manuscript.

## Conflict of Interest

The authors declare that the research was conducted in the absence of any commercial or financial relationships that could be construed as a potential conflict of interest.

## Publisher’s Note

All claims expressed in this article are solely those of the authors and do not necessarily represent those of their affiliated organizations, or those of the publisher, the editors and the reviewers. Any product that may be evaluated in this article, or claim that may be made by its manufacturer, is not guaranteed or endorsed by the publisher.
