# Corrigendum: Chemical Stimulants and Stressors Impact the Outcome of Virus Infection and Immune Gene Expression in Honey Bees (*Apis mellifera*)

**DOI:** 10.3389/fimmu.2022.844882

**Published:** 2022-03-02

**Authors:** Fenali Parekh, Katie F. Daughenbaugh, Michelle L. Flenniken

**Affiliations:** ^1^ Department of Microbiology and Immunology, Montana State University, Bozeman, MT, United States; ^2^ Department of Plant Sciences and Plant Pathology, Montana State University, Bozeman, MT, United States; ^3^ Pollinator Health Center, Montana State University, Bozeman, MT, United States

**Keywords:** honey bee, *Apis mellifera*, insect antiviral defense, honey bee viruses, deformed wing virus, thymol, fumagillin, clothianidin

In the original article, there was a mistake in **Figures 1**, **3**, **4**, **5**, **6** and their respective legends as published. **The original figures and figure legends, were mislabeled with “0.16 ppm thyme oil”. They should have been labeled with the correct label “0.16 ppb thyme oil” in all instances.** The correct figures and figure legends appears below.

**Figure 1:**
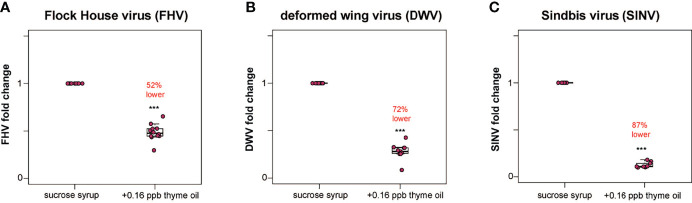
Lower virus abundance in honey bees fed thyme oil augmented sucrose syrup. Virus abundance in individual honey bees that were either fed sucrose syrup only or sucrose syrup augmented with 0.16 ppb thyme oil was assessed at 72 h post-infection by qPCR (n = 9-12 per treatment group) and the relative virus abundance is presented as ranked fold-changes. Together, these data illustrate that virus-infected bees fed thyme oil augmented sucrose syrup harbored less virus than virus-infected bees fed only sucrose syrup. **(A)** Flock House virus (FHV)-infected bees fed sucrose syrup augmented with thyme oil (0.16 ppb) had 52% less FHV (0.48 mean fold change) than bees fed sucrose syrup alone (p = 2.1 x 10^-5^). **(B)** In deformed wing virus (DWV)-infected bees, virus abundance was 72% less in bees fed sucrose syrup augmented with thyme oil (0.28 mean fold change) compared to bees fed only sucrose syrup (p = 0.00016). **(C)** Sindbis virus (SINV)-infected bees fed thyme oil augmented sucrose syrup had 87% less SINV (0.13 mean fold change) than bees fed sucrose syrup. Data were analyzed by a pairwise Wilcoxon Rank Sums with a Benjamini–Hochberg correction for multiple comparisons. Asterisks indicate a significant difference in virus abundance; significance levels: ***p < 0.0005. This figure includes results from one representative biological replicate for each virus (i.e., rep1). The data for all three biological replicates are presented in **Supplemental Figure S2** and raw data are in **Supplemental Tables S3, S4**.

**Figure 3:**
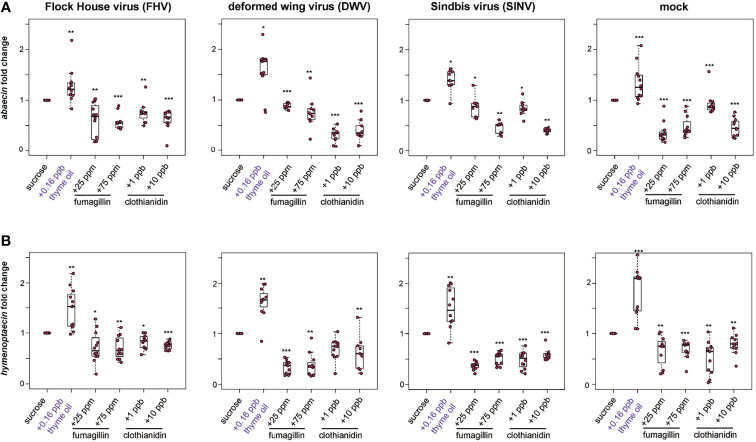
Expression of key RNAi genes was higher in honey bees fed thyme oil augmented sucrose syrup and lower in bees fed sucrose syrup containing fumagillin or clothianidin. Expression of *dicer-like* and *argonaute-2* in honey bees that were either mock- or virus-infected (i.e., FHV, DWV, SINV) and fed sucrose syrup only or syrup containing either thyme oil (0.16 ppb), fumagillin (25 ppm or 75 ppm), or clothianidin (1 ppb or 10 ppb) was assessed by qPCR. The ΔΔCt method with normalization to *rpl8* in mock or virus-infected bees fed sucrose syrup diet was utilized to determine the relative gene expression. **(A)** *Dicer-like* (*dcr-like*) expression was higher in virus-infected (i.e., FHV, DWV, SINV) and mock-infected bees fed thyme oil containing sucrose syrup, and lower in bees fed sucrose syrup containing fumagillin (25 ppm or 75 ppm) or clothianidin (1 ppb or 10 ppb) relative to bees fed sucrose syrup. **(B)** *Argonaute-2* (*ago2*) expression was higher in virus infected (i.e., FHV, DWV, SINV) or mock-infected bees fed thyme oil augmented sucrose syrup and reduced in bees fed diets containing fumagillin (25 ppm or 75 ppm) or clothianidin (1 ppb or 10 ppb), except in mock-infected bees fed 75 ppm fumagillin, *ago2* expression was similar to expression levels in bees fed sucrose only. Data were analyzed by a pairwise Wilcoxon Rank Sums with a Benjamini–Hochberg correction for multiple comparisons. Asterisks indicate a significant change in gene expression compared to sucrose only control; significance levels: *p < 0.05; **p < 0.005; ***p < 0.0005. This figure shows representative biological replicate for each gene (i.e., rep2 for *dcr-like* and rep3 for *ago2*). The data for all three biological replicates are presented in **Supplemental Figures S4, S5**. Raw data are included in **Supplemental Tables S7–S10**.

**Figure 4:**
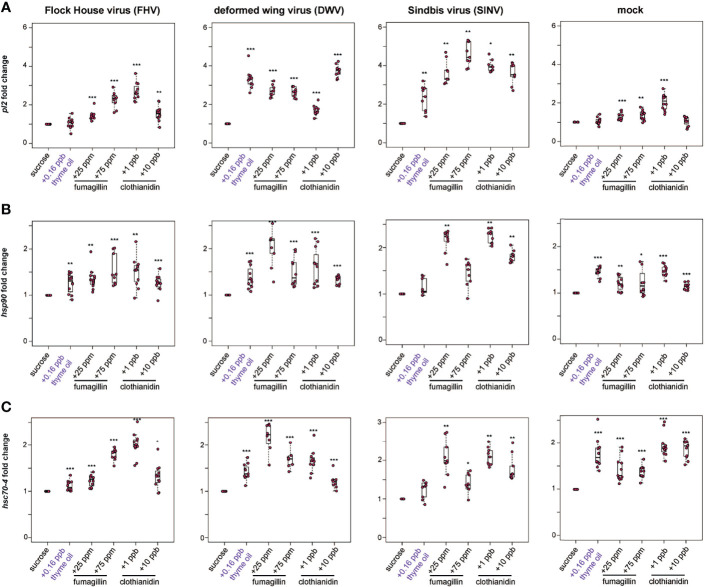
Expression of heat shock protein encoding genes was higher in honey bees fed sucrose syrup containing thyme oil, fumagillin, or clothianidin. The relative expression of three genes encoding heat shock proteins (*pl2, hsp90, hsc70-4*) was assessed using qPCR in mock or virus-infected bees fed sucrose syrup only or sucrose syrup containing additivities (i.e., thyme oil, fumagillin, or clothianidin). The ΔΔCt method with normalization to *rpl8* in mock or virus-infected bees fed sucrose syrup diet was utilized to determine the relative gene expression. **(A)** *Protein lethal(2)essential for life-like* (*pl2*) expression in virus-infected bees (i.e., FHV, DWV, SINV) fed sucrose syrup containing thyme oil (0.16 ppb), fumagillin (25 ppm or 75 ppm) or clothianidin (1 ppb or 10 ppb) was higher compared to expression in bees fed sucrose only. In mock-infected bees, *pl2* expression in bees fed sucrose syrup containing stimulant (0.16 ppb thyme oil) or 10 ppb clothianidin was similar to expression levels in bees fed sucrose only, whereas *pl2* expression was higher in bees fed sucrose syrup containing fumagillin (25 ppm or 75 ppm) or 1 ppb clothianidin. **(B)** *Heat shock protein 90* (*hsp90*) expression was higher in majority of the treatment groups, including virus- or mock-infected infected bees fed augmented sucrose syrups compared to bees fed non-augmented sucrose syrup except in SINV-infected bees fed thyme oil or 75 ppm fumagillin, which had similar *hsp90* expression levels to bees fed sucrose only. **(C)** *Heat shock 70-kDa protein cognate 4* (*hsc70-4*) expression was higher in the majority of treatment groups including mock and virus-infected bees fed sucrose syrup containing either thyme oil (0.16 ppb), fumagillin (25 ppm or 75 ppm) or clothianidin (1 ppb or 10 ppb), except in SINV-infected bees fed sucrose syrup containing thyme oil, which had *hsc70-4* expression levels similar to the controls. Data were analyzed by a pairwise Wilcoxon Rank Sums with a Benjamini–Hochberg correction for multiple comparisons. Asterisks indicate a significant change in gene expression compared to sucrose only control; significance levels: *p < 0.05; **p < 0.005; ***p < 0.0005. This figure shows representative biological replicate for *pl2* expression (i.e., rep1). The data for all three biological replicates for *pl2* are presented in **Supplemental Figure S6** and raw data are included in **Supplemental Tables S11–S16**.

**Figure 5:**
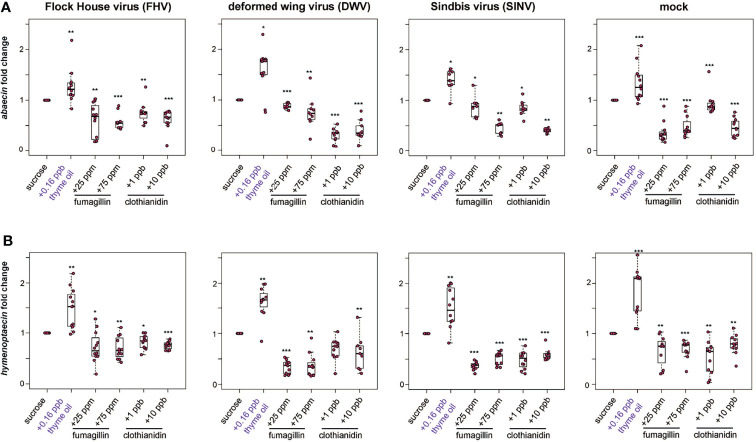
Expression of antimicrobial peptides encoding genes was greater in bees fed thyme oil augmented sucrose syrup and lower in bees fed fumagillin or clothianidin containing sucrose syrup. The expression of two antimicrobial peptides (*abaecin* and *hymenoptaecin*) was assessed in mock and virus-infected bees fed sucrose syrup containing additives. **(A)** In virus- and mock-infected bees (i.e., FHV, DWV, SINV), *abaecin* expression was higher in bees fed sucrose syrup containing thyme oil and lower in bees fed sucrose syrup containing fumagillin (25 ppm or 75 ppm) or clothianidin (1 ppb or 10 ppb) compared to bees fed sucrose only. **(B)** In virus-infected bees (i.e., FHV, DWV, SINV), *hymenoptaecin* expression was higher in bees fed sucrose syrup containing thyme oil and lower in bees fed sucrose syrup containing fumagillin (25 ppm or 75 ppm) or clothianidin (1 ppb or 10 ppb). *Hymenoptaecin* expression was higher in mock-infected bees fed sucrose syrup augmented with 0.16 ppb thyme oil, but lower in mock-infected bees fed either fumagillin (i.e., 25 ppm or 75 ppm) or clothianidin (1 ppb or 10 ppb) containing sucrose syrup. Data were analyzed by a pairwise Wilcoxon Rank Sums with a Benjamini–Hochberg correction for multiple comparisons. Asterisks indicate a significant change in gene expression compared to sucrose only control; significance levels: *p < 0.05; **p < 0.005; ***p < 0.0005. This figure shows representative biological replicate for the expression of each gene (i.e., rep1 for *abaecin* and rep3 for *hymenoptaecin*). The data for one additional biological replicate is presented in **Supplemental Figures S7, S8**. Raw data are included in **Supplemental Tables S17–S20**.

**Figure 6:**
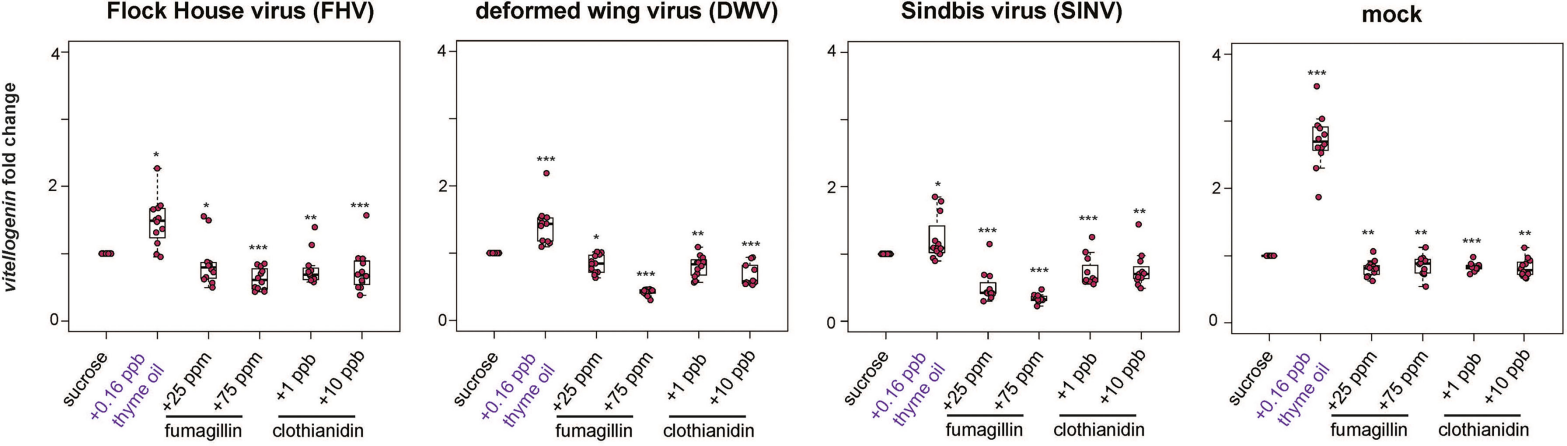
*Vitellogenin* expression was higher in bees fed sucrose syrup containing thyme oil and lower in bees fed sucrose syrup containing fumagillin or clothianidin. *Vitellogenin* expression was assessed using qPCR and the relative gene expression was analyzed using the ΔΔCt method with normalization to *rpl8* and relative to expression in mock or virus-infected bees fed sucrose syrup only. In virus-infected (i.e., FHV, DWV, SINV) and mock-infected honey bees *vitellogenin* expression was greater in bees fed sucrose syrup containing thyme oil 0.16 ppb and reduced in bees fed sucrose syrup containing fumagillin (25 ppm or 75 ppm) or clothianidin (1 ppb or 10 ppb) relative to sucrose only fed bees. Asterisks indicate a significant change in gene expression compared to sucrose only control; significance levels: *p < 0.05; **p < 0.005; ***p < 0.0005. This figure shows representative biological replicate for *vitellogenin* expression. The data for one additional biological replicate is presented in **Supplemental Figure S9**. Raw data are included in **Supplemental Tables S21, S22**.

In the original article, there was a mistake in the **Supplementary Material** as published. **Throughout the Supplementary Material “0.16 ppm thyme oil” should be corrected to “0.16 ppb thyme oil” in all instances.** The correct files appear below.

In the original article, there was an error. **Throughout the text it was mistakenly written that “0.16 ppm thyme oil” was utilized for experiments.** It should be written “0.16 ppb thyme oil” for all experiments. The original article has been updated.

In addition, in the original article, there was an error**. It was mistakenly written “0.06 ppm thymol” instead of “0.06 ppb thymol”**: “We estimate that 0.16 ppb thyme oil used in this experiment may contain approximately 0.06 ppb (60 ppb) thymol (37% thymol in 0.16 ppb thyme oil corresponds to ~ 60 ppb)”.

A correction has been made to **4. Materials and Methods, 4.4. Honey Bee Diet Preparation:** “Post-injection, honey bees were housed in modified deli containers for the duration of the study. Bees in the control group were fed 50% sucrose syrup only, whereas bees in treatment groups were fed sucrose syrup containing one of the following additives: 0.16 ppb thyme oil (Body wonders), Fumagilin-B^®^, fumagillin dicyclohexyl ammonium (Medivet Pharmaceuticals Ltd.) at manufacturer’s recommended dose of 25 ppm or a higher dose of 75 ppm, or clothianidin at the field relevant sublethal concentration of 1 ppb or near lethal dose of 10 ppb (130, 195, 196). Thyme oil contains 10% - 64% thymol (~37% average) depending on the plant species, geographical sources, and harvest season, which may affect the volatile composition of the plant (113, 116-118). We estimate that 0.16 ppb thyme oil used in this experiment may contain approximately 0.06 ppb thymol (37% thymol in 0.16 ppb thyme oil corresponds to ~ 0.06 ppb). For clothianidin treatments we utilized the commercially available Poncho^®^ 600, which contains 48% of the active ingredient clothianidin. A working stock of 1000 ppb clothianidin was prepared by 1:10 serial dilutions in 50% sucrose solution, which was further diluted in 50% sucrose to prepare 1 ppb (i.e., 10 ul of 1000 ppb Poncho^®^ 600) and 10 ppb (i.e., 100 ul of 1000 ppb Poncho^®^ 600) clothianidin solutions. Honey bees were fed sucrose solution either alone or with additives using cage feeders that were made by putting two holes on each side of a 1.5 mL centrifuge tube; sucrose solution was checked daily and refilled as needed throughout the study.”

The authors apologize for these errors and state they do not change the scientific conclusions of the article in any way.

## Publisher’s Note

All claims expressed in this article are solely those of the authors and do not necessarily represent those of their affiliated organizations, or those of the publisher, the editors and the reviewers. Any product that may be evaluated in this article, or claim that may be made by its manufacturer, is not guaranteed or endorsed by the publisher.

