# Supplementary material for: Chemical Stimulants and Stressors Impact the Outcome of Virus Infection and Immune Gene Expression in Honey Bees (Apis mellifera)
Source: Front Immunol. 2021 Oct 28;12:747848. doi: 10.3389/fimmu.2021.747848 (PMC8596368; doi:10.3389/fimmu.2021.747848)
Supplement: Supplementary file 1 [file DataSheet_1.pdf]

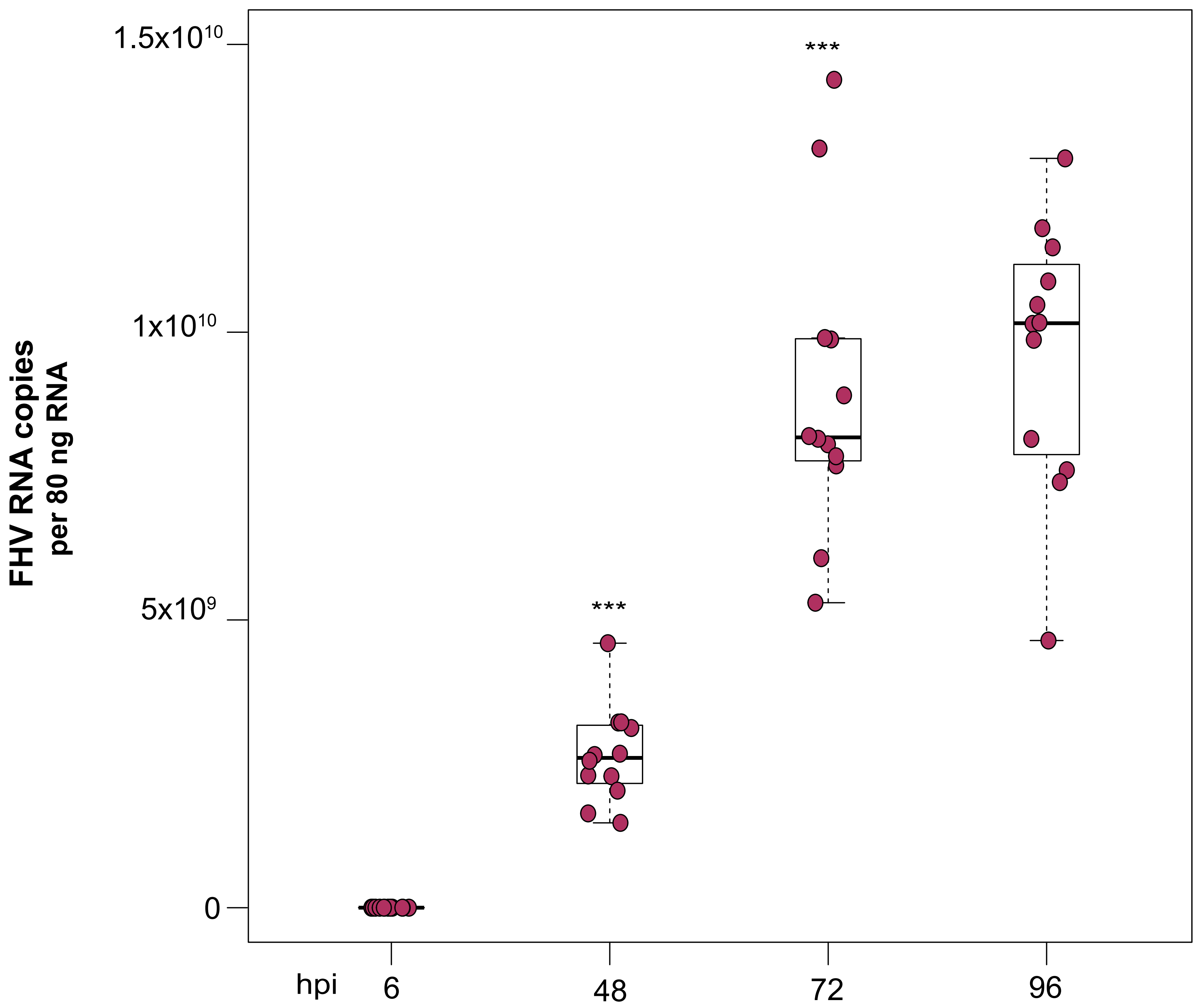

Supplemental Figure S1. Increased virus abundance in FHV-infected bees over time.

FHV abundance in individual honey bees injected with 3.5 x 10^8 GE FHV/bee was assessed over a time course (i.e., 6 hpi, 48 hpi, 72 hpi, 96 hpi) by qPCR (n= 9-12 per treatment group). FHV abundance was 8,130 fold greater at 48 hpi (p= 1.5x10^{-6}) and 27,526 fold greaterat 72 hpi (p= 1.5x10^{-6}) relative to 6 hpi. FHV levels at 72 hpi and 96 hpi were similar (p = 0.41). Statistical significance was assessed using Wilcoxon Ranks Sums test. Asterisks indicate a significant difference in virus abundance compared to 6 hpi, significance levels:

*** $p < 0.0005$.

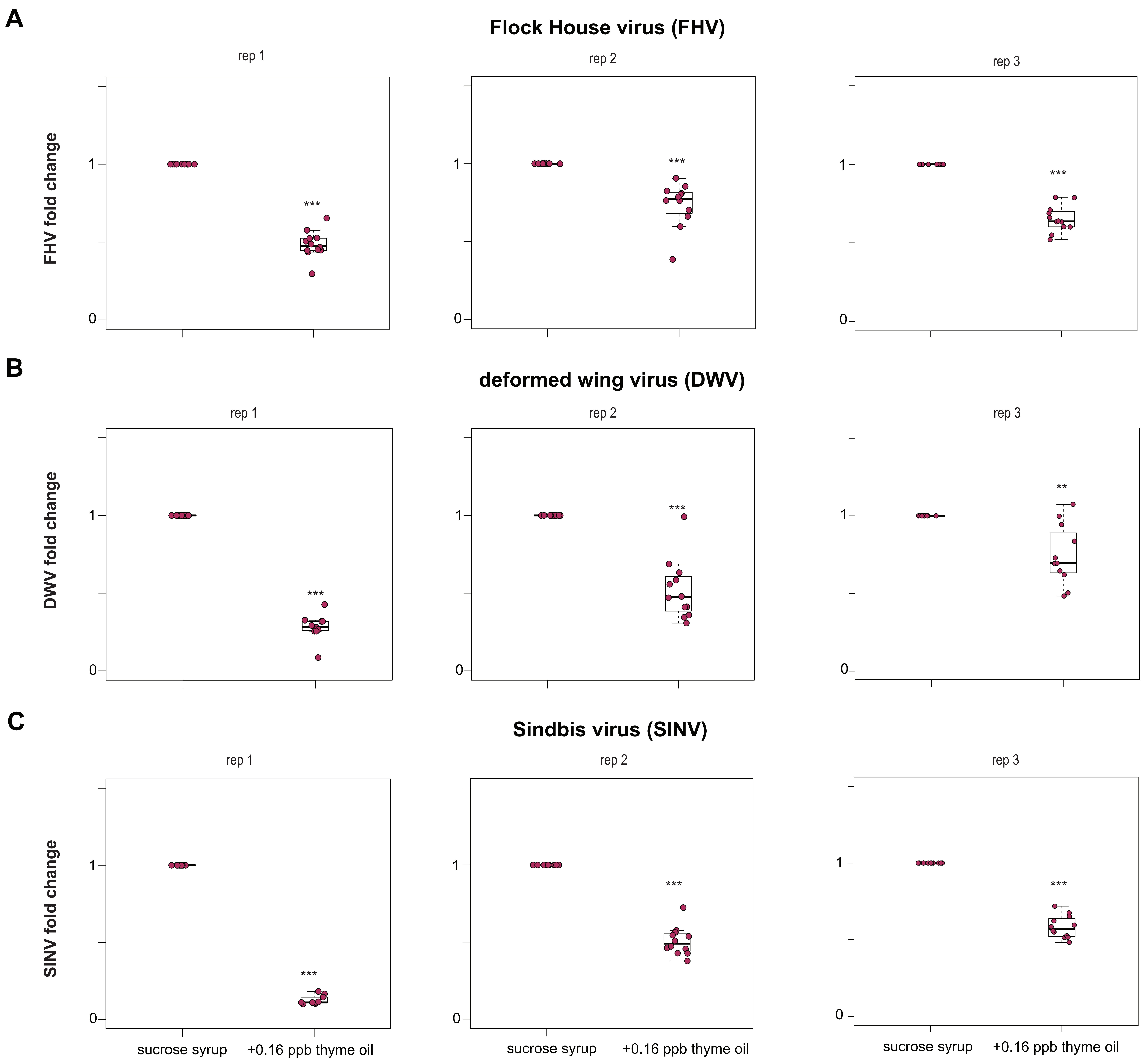

Supplemental Figure S2. Lower virus abundance in honey bees fed thyme oil augmented sucrose syrup in three biological replicates. Virus (FHV, DWV, SINV) abundance in individual honey bees at 72 h post-infection was assessed by qPCR (n= 9-12 per treatment group) in each of three experimental replicates (rep1, rep2, and rep3), one of which was also included in Figure 1. **(A)** FHV-infected bees fed 0.16 ppb thyme oil augmented sucrose syrup harbored less virus than FHV-infected bees fed sucrose syrup only in all three replicates. **(B)** DWV-infected bees fed 0.16 ppb thyme oil augmented sucrose syrup harbored less virus than virus-infected bees fed sucrose syrup only in three replicates of the experiment. **(C)** SINV-infected bees had lower virus abundance when fed sucrose syrup augmented with 0.16 ppb thyme oil relative to bees fed sucrose syrup in all three replicates. Asterisks indicate a significant change in virus abundance compared to the control groups (i.e., virus-infected bees fed sucrose syrup only); significance levels: ** $p < 0.005$; *** $p < 0.0005$.

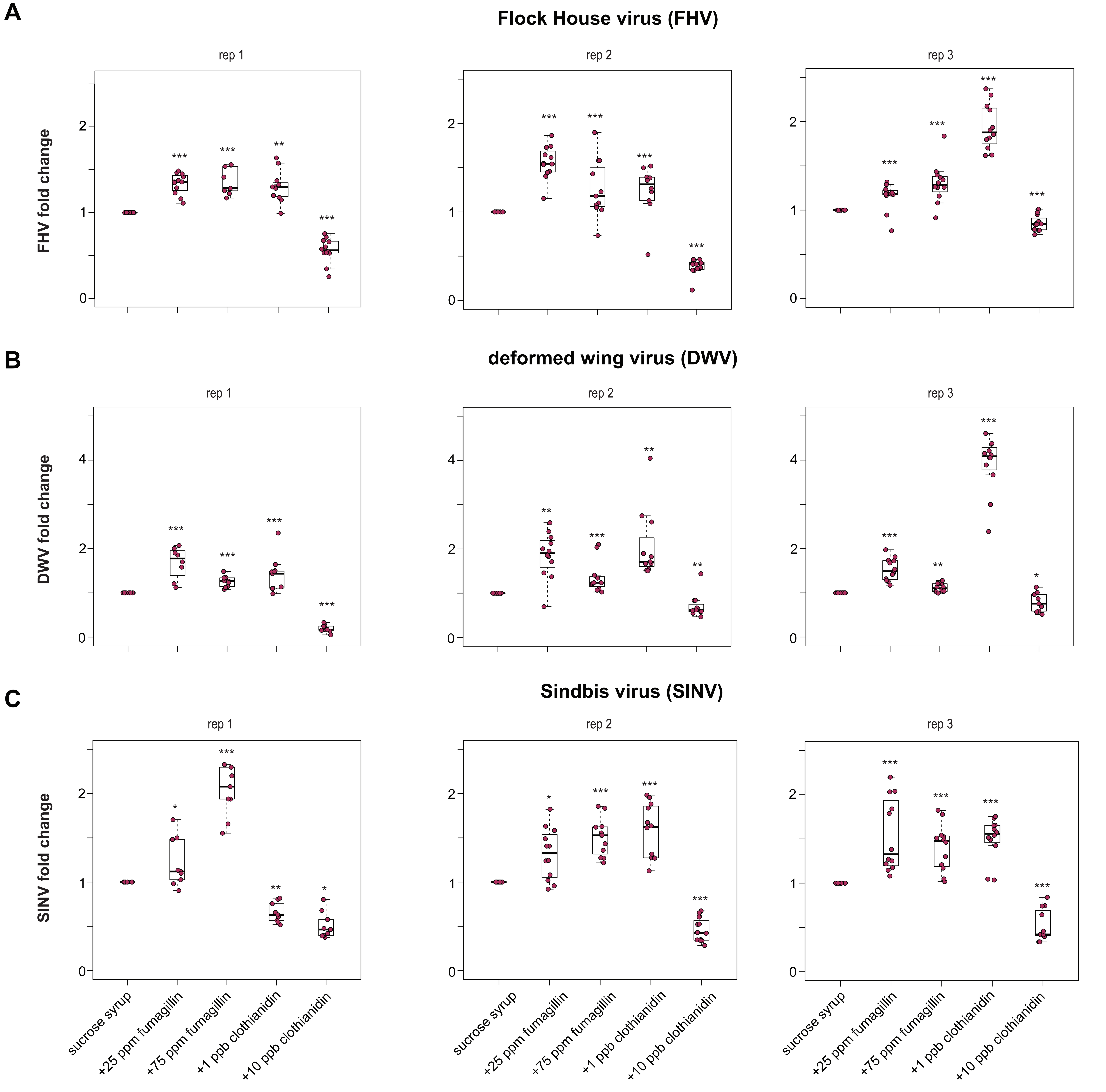

Supplemental Figure S3. Honey bees fed sucrose syrup containing fumagillin or clothianidin had greater virus levels than bees feed only sucrose syrup in three biological replicates.

Virus (FHV, DWV, SINV) abundance in individual honey bees at 72 h post-infection was assessed by qPCR (n= 9-12 per treatment group) in each of three experimental replicates (rep1, rep2, and rep3), one of which was also included in Figure 2. **(A)** FHV-infected bees fed fumagillin (25 ppm or 75 ppm), or 1 ppb clothianidin containing sucrose syrup harbored more virus than FHV-infected bees fed sucrose syrup only. FHV-infected bees fed 10 ppb clothianidin containing syrup had lower virus abundance compared to FHV-infected bees fed sucrose syrup in three replicates. **(B)** DWV-infected bees fed sucrose syrup containing fumagillin (25 ppm or 75 ppm), or 1 ppb clothianidin had higher virus abundance, whereas bees fed 10 ppb clothianidin containing syrup had lower virus abundance compared to DWV-infected bees fed sucrose syrup in all three replicates. **(C)** SINV levels were greater in SINV-infected bees fed sucrose syrup containing fumagillin (25 ppm or 75 ppm) or 1 ppb clothianidin, whereas bees fed 10 ppb clothianidin containing syrup had lower virus abundance compared to SINV-infected bees fed sucrose syrup. Asterisks indicate a significant change in virus abundance compared to the control groups (i.e., virus-infected bees fed sucrose syrup only); significance levels: * $p < 0.05$; ** $p < 0.005$; *** $p < 0.0005$.

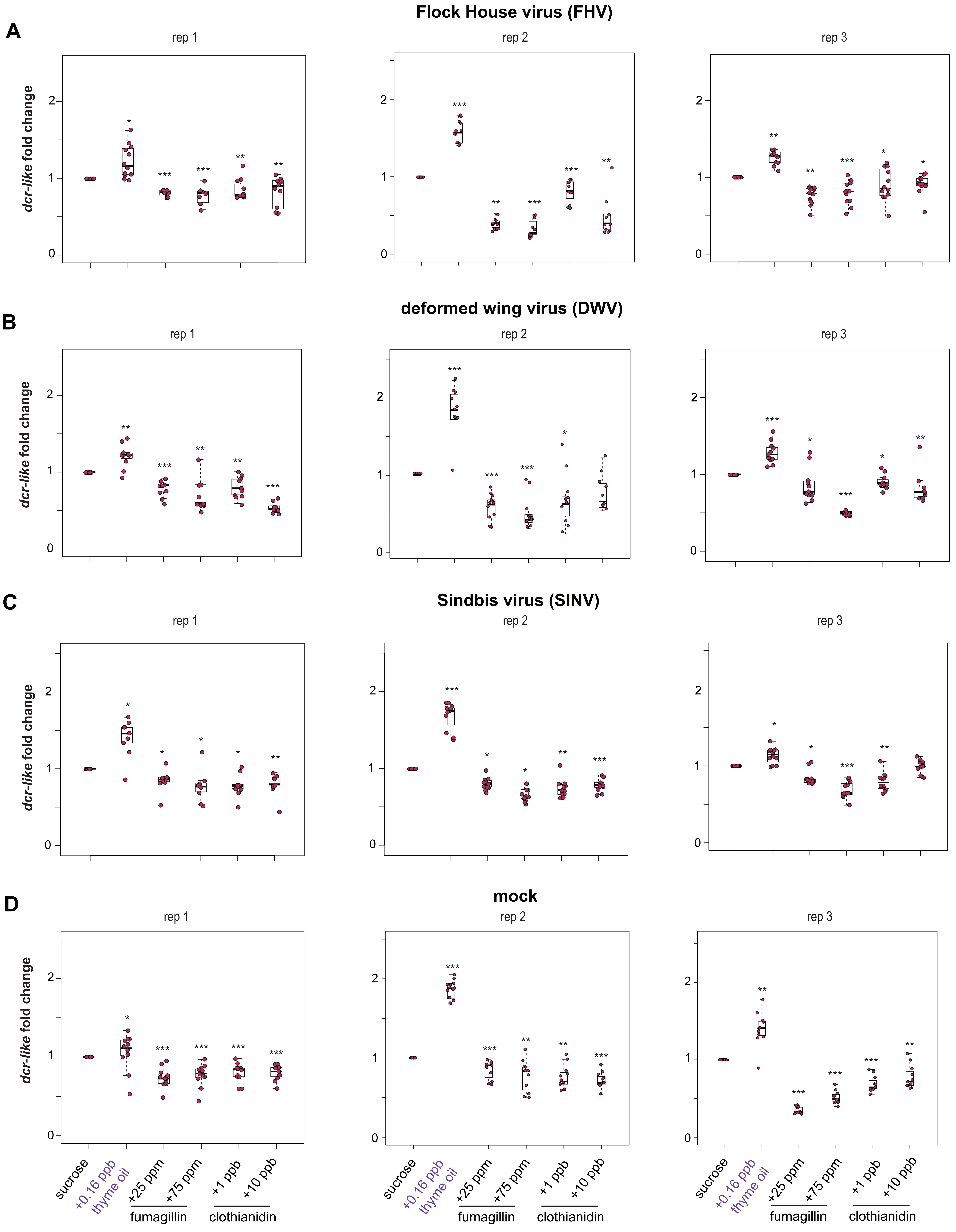

Supplemental Figure S4. Expression of *dicer-like* was higher in bees fed thyme oil augmented sucrose syrup, whereas it was reduced in bees fed sucrose syrup containing fumagillin or clothianidin
The expression of *dcr-like* in honey bees that were either mock- or virus-infected and fed sucrose syrup only or syrup containing either thyme oil (0.16 ppb), fumagillin (25 ppm or 75 ppm), or clothianidin (1 ppb or 10 ppb) was assessed by qPCR. The relative expression of *dcr-like* was evaluated using the ΔΔCt method. Specifically, *dcr-like* expression relative to the *rpl8* housekeeping gene was determined (ΔCt), and then expression of individuals in each treatment group was compared to either virus-infected (A-C) or mock-infected (D) honey bees fed sucrose syrup only. **(A, B, C)** *Dcr-like* expression was higher in virus-infected bees (i.e., FHV, DWV, SINV) fed 0.16 ppb thyme oil augmented sucrose syrup and reduced in virus-infected bees fed fumagillin (25 ppm or 75 ppm) and clothianidin (1 ppb or 10 ppb) containing sucrose syrup compared to virus-infected bees fed sucrose syrup only. **(D)** The expression of *dcr-like* was higher in mock-infected bees fed 0.16 ppb thyme oil augmented sucrose syrup compared to bees fed sucrose syrup only. In contrast, *dcr-like* expression was lower in mock-infected bees fed sucrose syrup containing fumagillin (25 ppm or 75 ppm) and clothianidin (1 ppb or 10ppb) compared to bees fed only sucrose syrup in three biological replicates. Asterisks indicate a significant change in gene expression compared to sucrose only control; significance levels: * $p < 0.05$; ** $p < 0.005$; *** $p < 0.0005$.

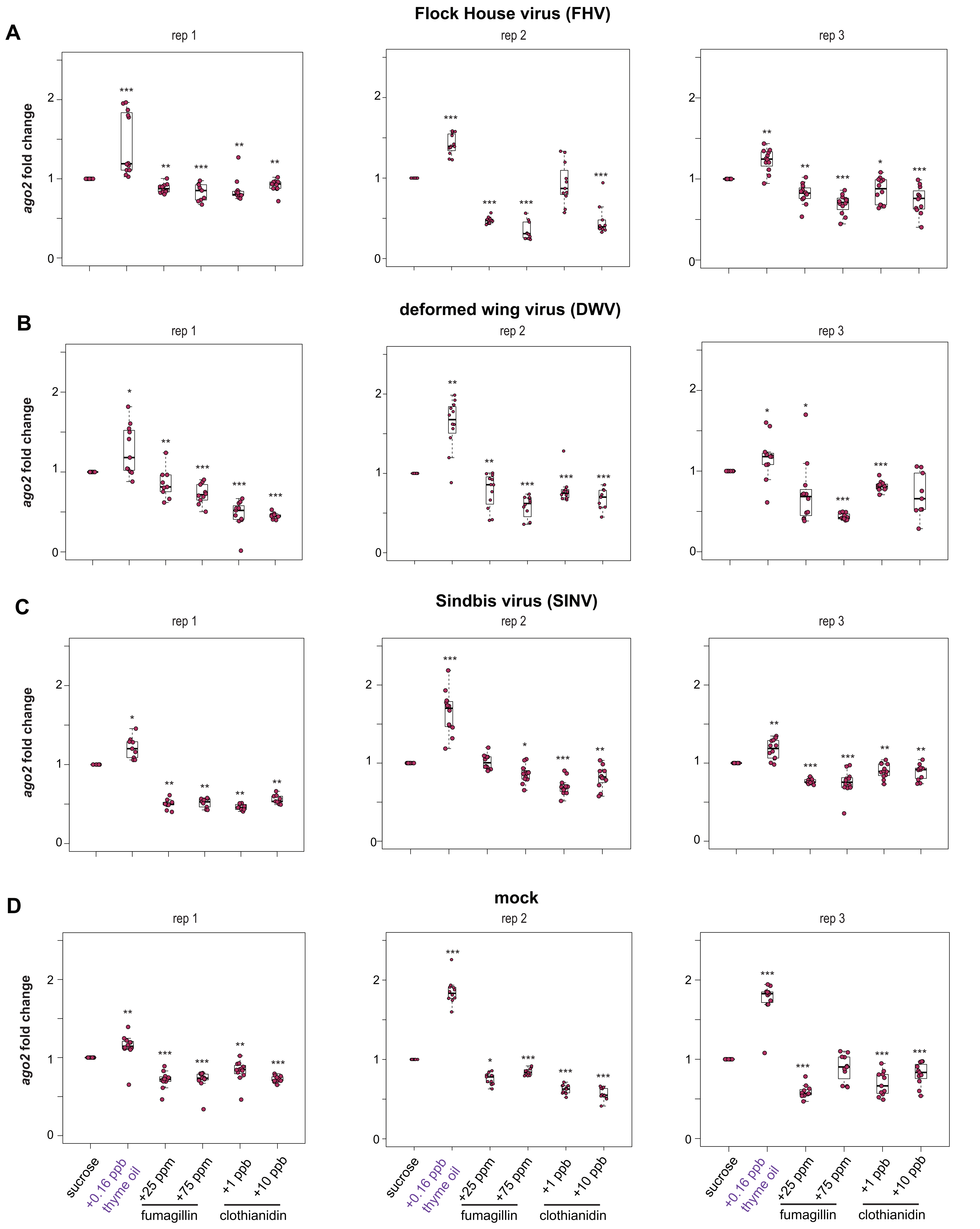

Supplemental Figure S5. Expression of *argonaute-2* was higher in honey bees fed thyme oil augmented sucrose syrup and lower in bees fed sucrose syrup containing fumagillin or clothianidin.
The expression of *ago2* in honey bees that were either mock- or virus-infected and fed sucrose syrup only or syrup containing either thyme oil (0.16 ppb), fumagillin (25 ppm or 75 ppm), or clothianidin (1 ppb or 10 ppb) was assessed by qPCR. The relative expression of *ago2* was evaluated using the ΔΔCt method. Specifically, *ago2* expression relative to the *rpl8* housekeeping gene was determined (ΔCt), and then expression of individuals in each treatment group was compared to either virus-infected (A-C) or mock-infected (D) honey bees fed sucrose syrup only. **(A, B, C)** The expression of *ago2* was consistently higher in virus-infected bees (i.e., FHV, DWV, SINV) fed thyme oil (0.16 ppb) augmented sucrose syrup, and lower in bees fed sucrose syrup containing fumagillin (25 ppm or 75 ppm) or clothianidin (1 ppb or 10 ppb). **(D)** The expression of *ago2* in mock-infected bees was higher in bees fed 0.16 ppb thyme oil augmented sucrose syrup compared to bees fed sucrose syrup only and lower in mock-infected bees fed diets containing fumagillin (25 ppm or 75 ppm) or clothianidin (1 ppb or 10 ppb) than bees fed only sucrose syrup. Asterisks indicate a significant change in gene expression compared to sucrose only control; significance levels: * $p < 0.05$; ** $p < 0.005$; *** $p < 0.0005$.

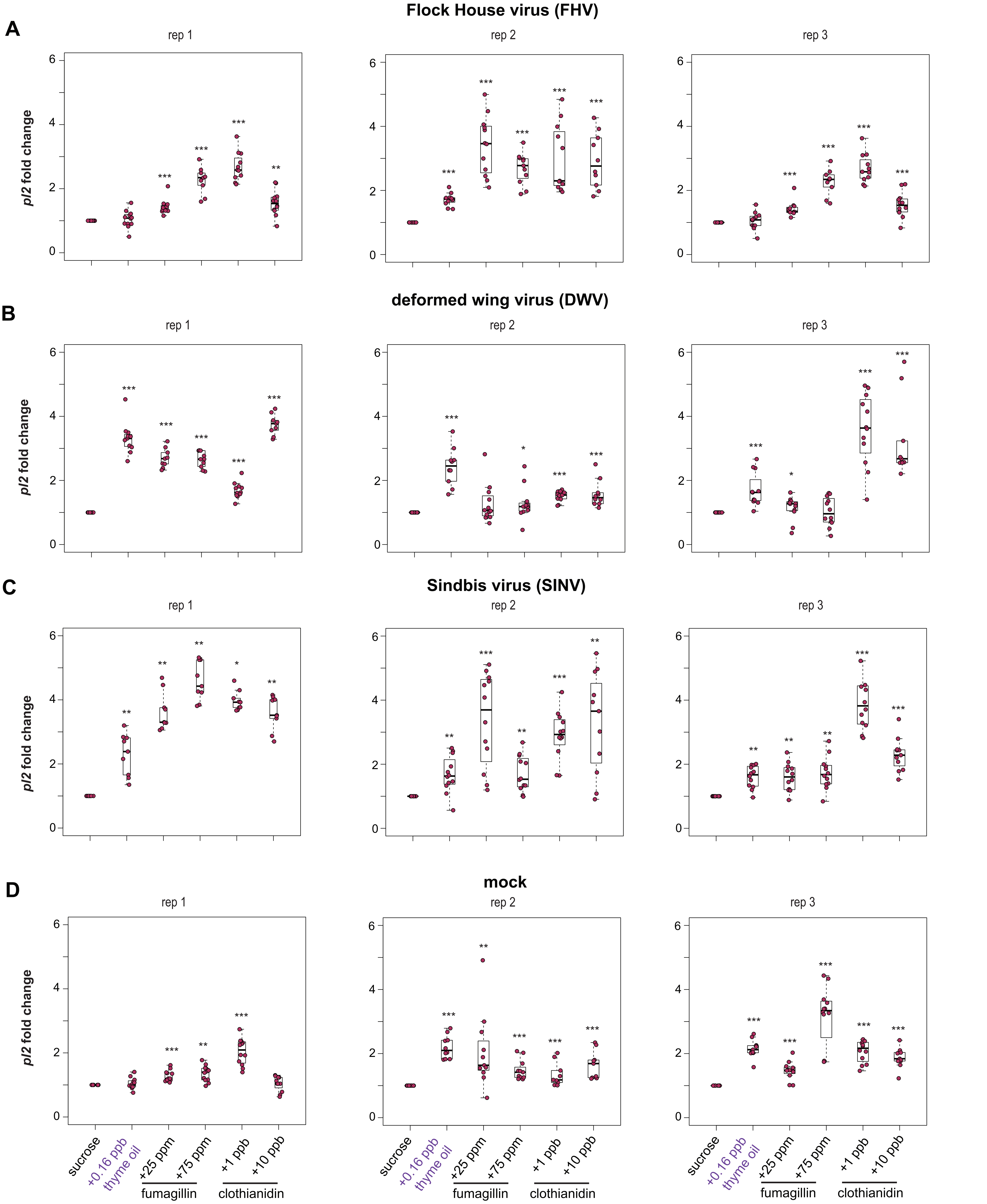

Supplemental Figure S6. Expression of *protein-essential for life-like-2 (pl2)* was higher in honey bees fed sucrose syrup containing thyme oil, fumagillin or clothianidin.
The expression of *pl2* in honey bees that were either mock- or virus-infected and fed sucrose syrup only or sucrose syrup containing either thyme oil (0.16 ppb), fumagillin (25 ppm or 75 ppm), or clothianidin (1 ppb or 10 ppb) was assessed by qPCR. The relative expression of *pl2* was evaluated using the ΔΔCt method. Specifically, *pl2* expression relative to the rpl8 housekeeping gene was determined (ΔCt), and then expression of individuals in each treatment group was compared to either virus-infected (A-C) or mock-infected (D) honey bees fed sucrose syrup only. **(A, B, C)** The expression level of *pl2* was higher in virus-infected bees (i.e., FHV, DWV, SINV) fed sucrose syrup containing thyme oil, fumagillin (25 ppm or 75 ppm) or clothianidin (1 ppb or 10 ppb) in three biological replicates. **(D)** The expression of *pl2* in mock-infected bees was higher in bees fed sucrose syrup containing 0.16 ppb thyme oil, fumagillin (25 ppm or 75 ppm) or clothianidin (1 ppb or 10 ppb) compared to bees fed sucrose syrup only. Asterisks indicate a significant change in gene expression compared to sucrose only control; significance levels: * $p < 0.05$; ** $p < 0.005$; *** $p < 0.0005$.

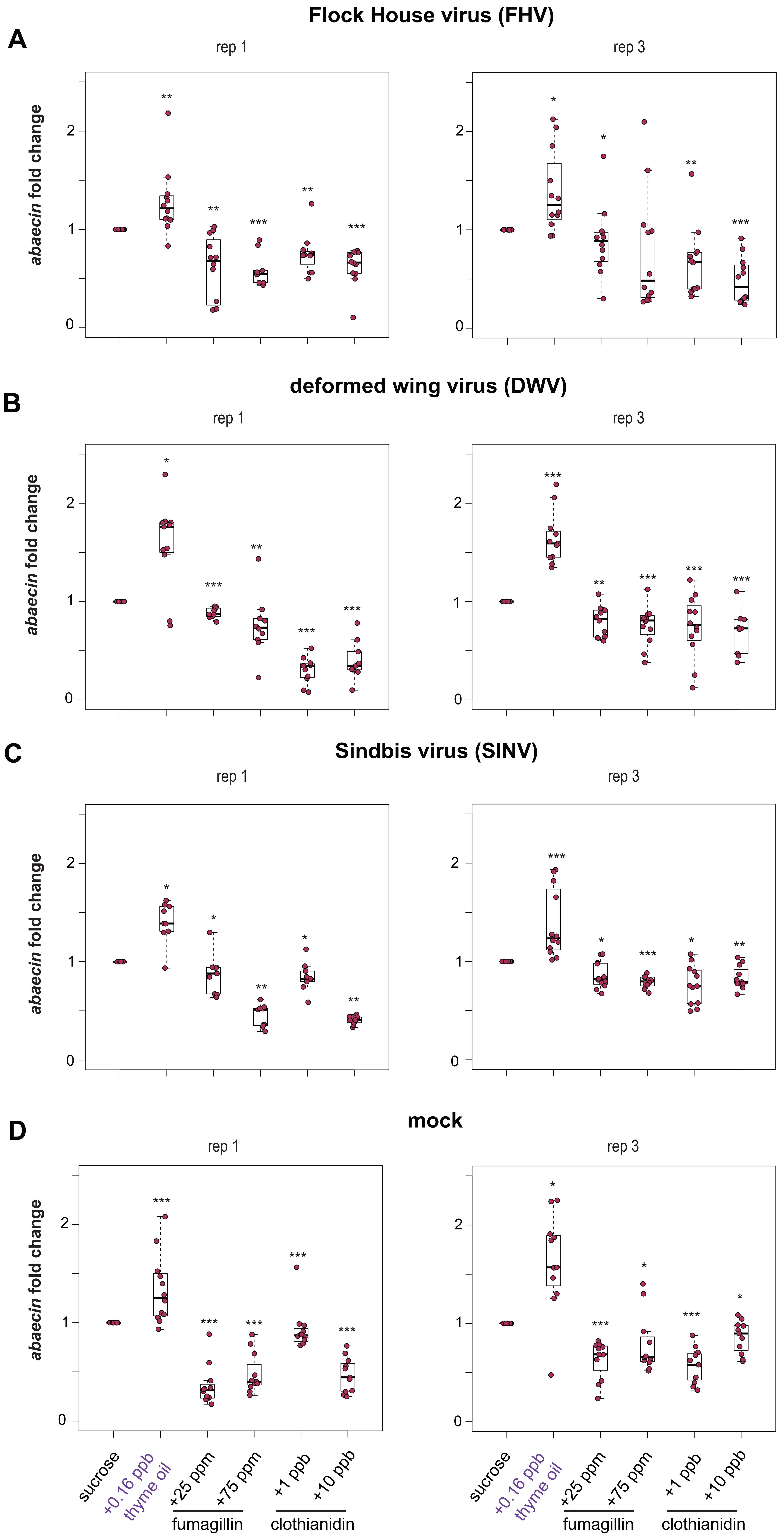

Supplemental Figure S7. *Abaecin* expression was higher in honey bees fed sucrose syrup containing thyme oil and lower in bees fed sucrose syrup containing fumagillin or clothianidin.

The expression of *abaecin* in honey bees that were either mock- or virus-infected and fed sucrose syrup only or sucrose syrup containing either thyme oil (0.16 ppb), fumagillin (25 ppm or 75 ppm), or clothianidin (1 ppb or 10 ppb) was assessed by qPCR. The relative expression of *abaecin* was evaluated using the ΔΔCt method. Specifically, *abaecin* expression relative to the *rpl8* housekeeping gene was determined (ΔCt), and then expression of individuals in each treatment group was compared to either virus-infected (A-C) or mock-infected (D) honey bees fed sucrose syrup only. **(A, B, C)** *Abaecin* expression was higher in virus-infected bees (i.e., FHV, DWV, SINV) fed sucrose syrup containing thyme oil, and lower in bees fed sucrose syrup containing fumagillin (25 ppm or 75 ppm) or clothianidin (1 ppb or 10 ppb). **(D)** *Abaecin* expression in mock-infected bees was higher in bees fed sucrose syrup containing 0.16 ppb thyme oil, and lower in sucrose syrup containing fumagillin (25 ppm or 75 ppm) or clothianidin (1 ppb or 10 ppb) compared to bees fed sucrose syrup only, in two biological replicates, except in rep3, *abaecin* expression in FHV-infected bees fed sucrose syrup containing 75 ppm fumagillin was similar to controls. Significance levels: * $p < 0.05$; ** $p < 0.005$; *** $p < 0.0005$.

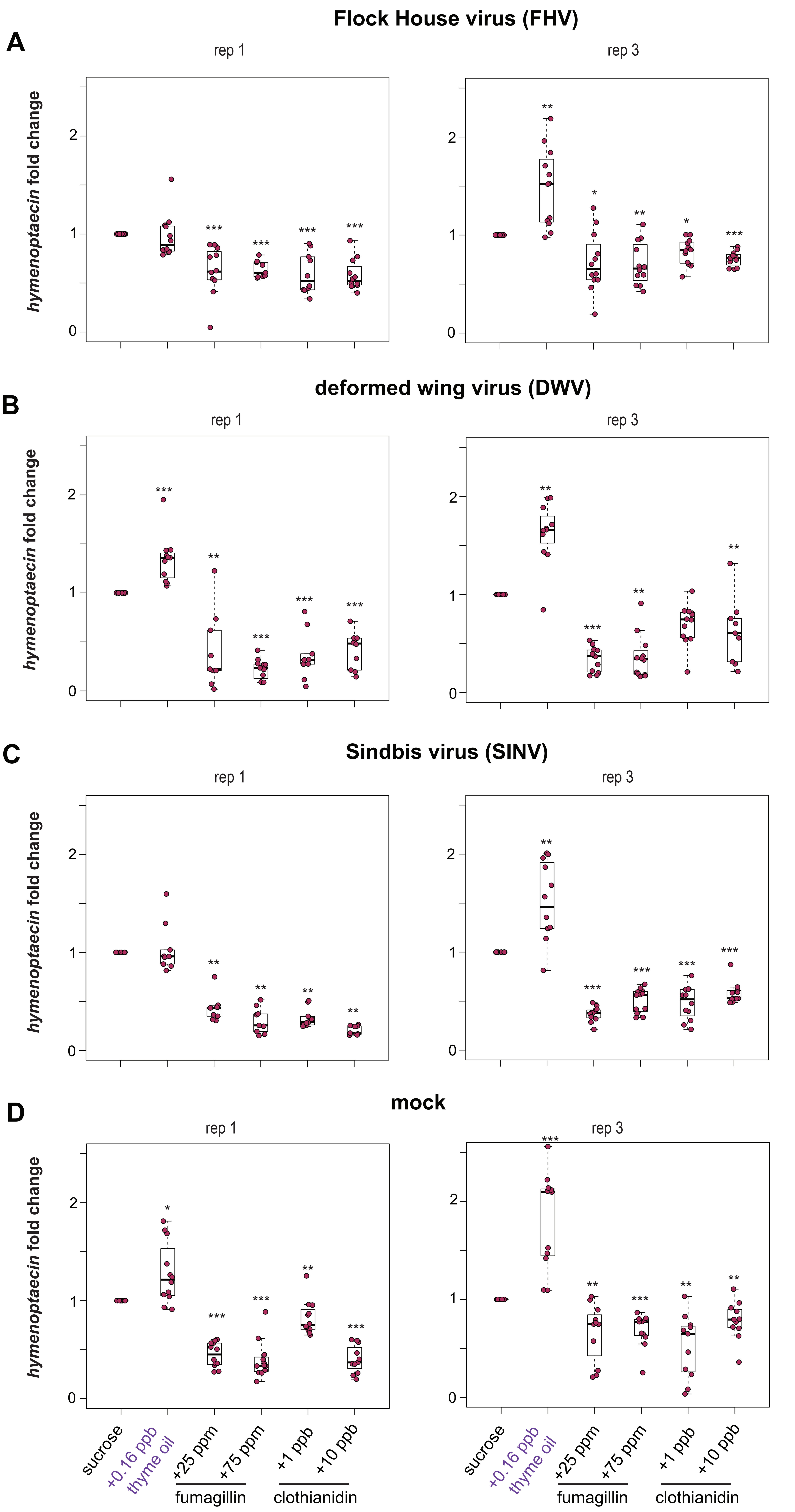

Supplemental Figure S8. Hymenoptaecin expression was higher in honey bees fed sucrose syrup containing thyme oil and lower in bees fed sucrose syrup containing fumagillin or clothianidin.
The expression of *hymenoptaecin* in honey bees that were either mock- or virus-infected and fed sucrose syrup only or sucrose syrup containing either thyme oil (0.16 ppb), fumagillin (25 ppm or 75 ppm), or clothianidin (1 ppb or 10 ppb) was assessed by qPCR. The relative expression of *hymenoptaecin* was evaluated using the ΔΔCt method. Specifically, *hymenoptaecin* expression relative to the *rpl8* housekeeping gene was determined (ΔCt), and then expression of individuals in each treatment group was compared to either virus-infected (A-C) or mock-infected (D) honey bees fed sucrose syrup only. **(A, B, C)** Higher *hymenoptaecin* expression was observed in virus-infected bees (i.e., FHV, DWV, SINV) fed sucrose syrup containing thyme oil, and lower in bees fed sucrose syrup containing fumagillin (25 ppm or 75 ppm) or clothianidin (1 ppb or 10 ppb), **(D)** In mock-infected bees, *hymenoptaecin* expression was higher in bees fed sucrose syrup containing 0.16 ppb thyme oil, and lower in sucrose syrup containing fumagillin (25 ppm or 75 ppm) or clothianidin (1 ppb or 10 ppb) compared to bees fed sucrose syrup only, in two biological replicates, except in rep1, FHV and SINV-infected bees fed sucrose syrup containing thyme oil, *hymenoptaecin* expression was similar to bees fed sucrose syrup. Asterisks indicate a significant change in gene expression compared to sucrose only control; significance levels: * $p < 0.05$; ** $p < 0.005$; *** $p < 0.0005$.

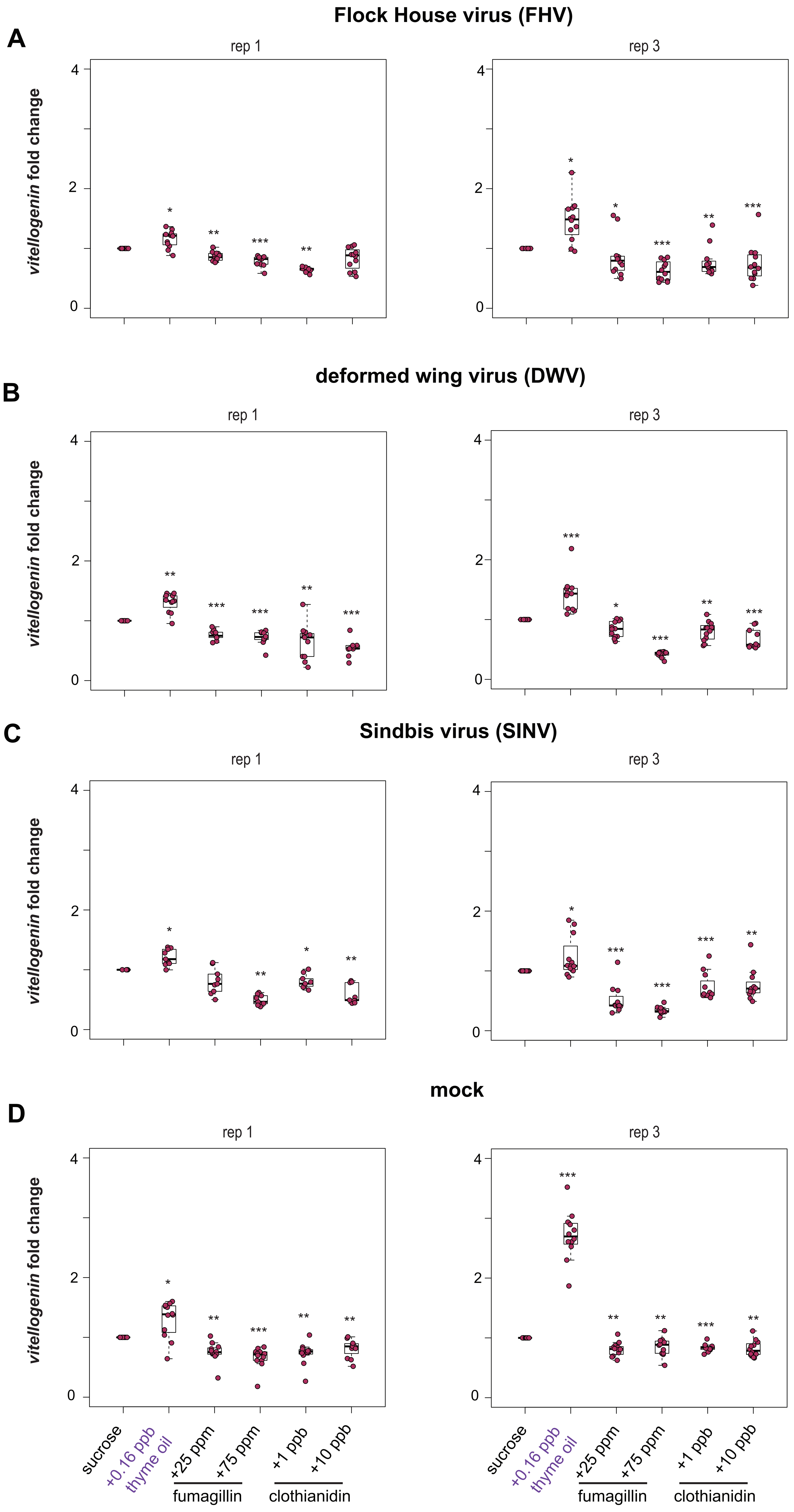

Supplemental Figure S9. ***Vitellogenin*** **expression was higher in bees fed sucrose syrup containing thyme oil and lower in bees fed sucrose syrup containing fumagillin or clothianidin.**

The expression of *vitellogenin* in honey bees that were either mock- or virus-infected and fed sucrose syrup only or sucrose syrup containing either thyme oil (0.16 ppb), fumagillin (25 ppm or 75 ppm), or clothianidin (1 ppb or 10 ppb) was assessed by qPCR. The relative expression of *vitellogenin* was evaluated using the ΔΔCt method. Specifically, *vitellogenin* expression relative to the *rpl8* housekeeping gene was determined (ΔCt), and then expression of individuals in each treatment group was compared to either virus-infected (A-C) or mock-infected (D) honey bees fed sucrose syrup only. **(A, B, C)** *vitellogenin* expression was higher in virus-infected bees (i.e., FHV, DWV, SINV) fed sucrose syrup containing thyme oil, and lower in bees fed sucrose syrup containing fumagillin (25 ppm or 75 ppm) or clothianidin (1 ppb or 10 ppb) compared to expression levels in bees fed only sucrose syrup. **(D)** In mock-infected bees, *vitellogenin* expression was higher in bees fed sucrose syrup containing 0.16 ppb thyme oil, and lower in sucrose syrup containing fumagillin (25 ppm or 75 ppm) or clothianidin (1 ppb or 10 ppb) compared to bees fed sucrose syrup only, in two biological replicates, except in rep1, FHV-infected bees fed 10 ppb clothianidin and SINV-infected bees fed 25 ppm fumagillin which had *vitellogenin* expression similar to bees fed only sucrose syrup. Asterisks indicate a significant change in gene expression compared to sucrose only control; significance levels: * $p < 0.05$; ** $p < 0.005$; *** $p < 0.0005$.

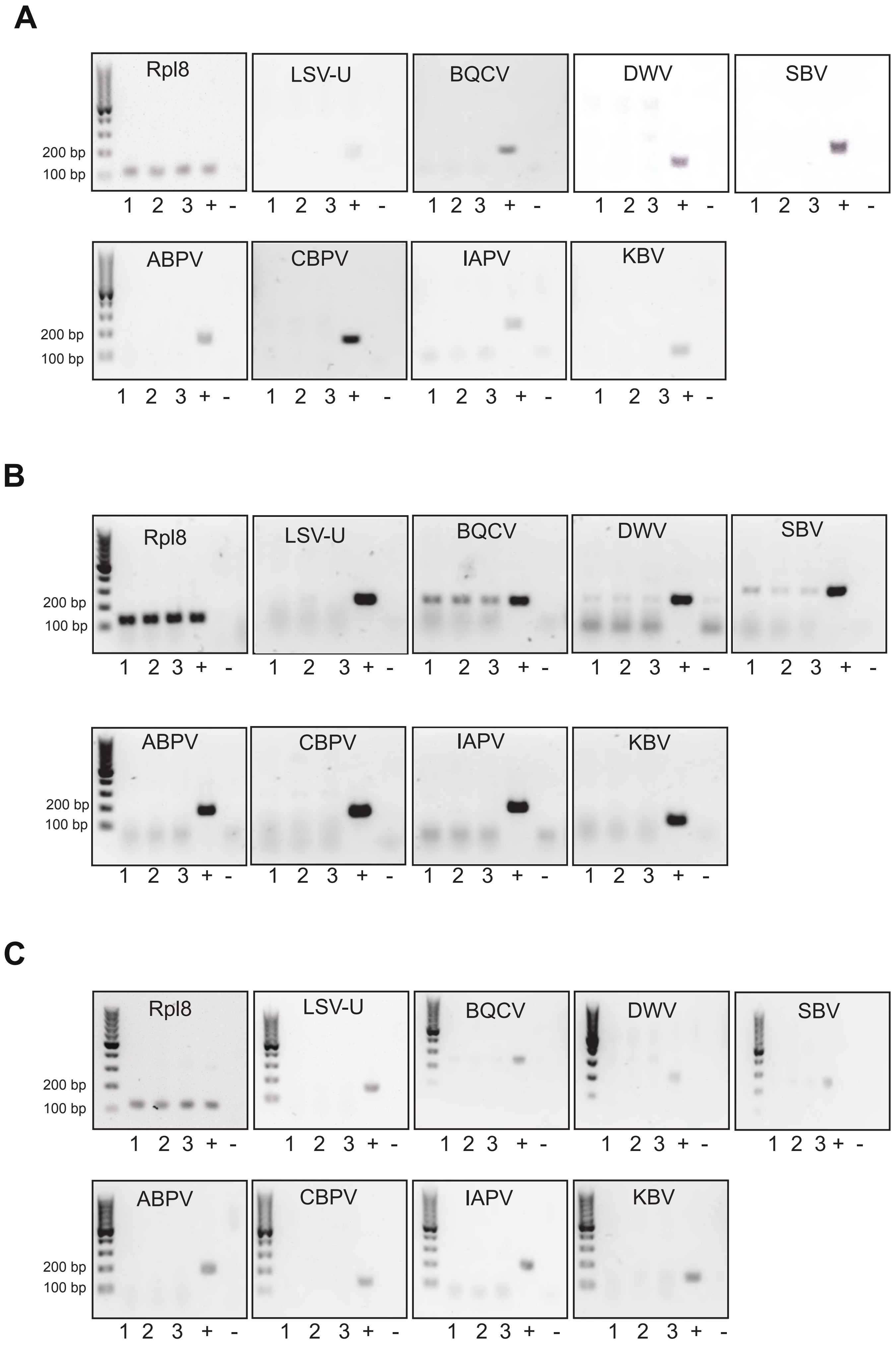

Supplemental Figure S10. Honey bee virus test results.
Virus-specific PCR was utilized to test for the presence of confounding, pre-existing infections in honey bees obtained from the same frame at the same time as the honey bees utilized for the experiments described herein. RNA was isolated from individual honey bee abdomens that were mock-infected and housed in identical conditions as the experimental bees for 72 h. The RNA was reverse transcribed, and the quality of the resulting cDNA was assessed using primers specific for honey bee housekeeping gene rpl8. Pathogen specific PCR was carried out to test the presence of the following viruses, using primers listed in Supplemental Table S1, LSV-U (Lake Sinai virus 1 and 2), BQCV (black queen cell virus), DWV (deformed wing virus), SBV (sacbrood virus), ABPV (acute bee paralysis virus), CBPV (chronic bee paralysis virus), IAPV (Israeli acute paralysis virus), and KBV (Kashmir bee virus). Pathogen testing was performed on pooled cDNA samples representing four individual bees (numbered pools 1, 2, and 3) and positive (+) and negative (-) controls were included for all tests. Amplicons were electrophoresed through a 1.5% agarose gel and stained with SYBR Safe. The results from template-containing positive controls and template-lacking negative controls were as expected. **(A)** Pathogen test results of pooled cDNA samples from biological replicate 1. The PCR results for these samples indicate that the levels of tested viruses in biological replicate 1 were below the limit of detection. **(B)** Pathogen test results of pooled cDNA samples from biological replicate 2. The PCR results indicate that the majority of virus levels in biological replicate 2 were below the limit of detection except for detection of low levels BQCV, DWV, and SBV, based on qualitative assessment of product band intensity relative to positive control. **(C)** Pathogen-specific PCR tests of pooled cDNA samples from biological replicate 3 indicate that the levels of tested in biological replicate 3 were below the limit of detection. Each gel image in panel A and panel B was aligned to the 100 bp DNA ladder on the first gel image in each row.

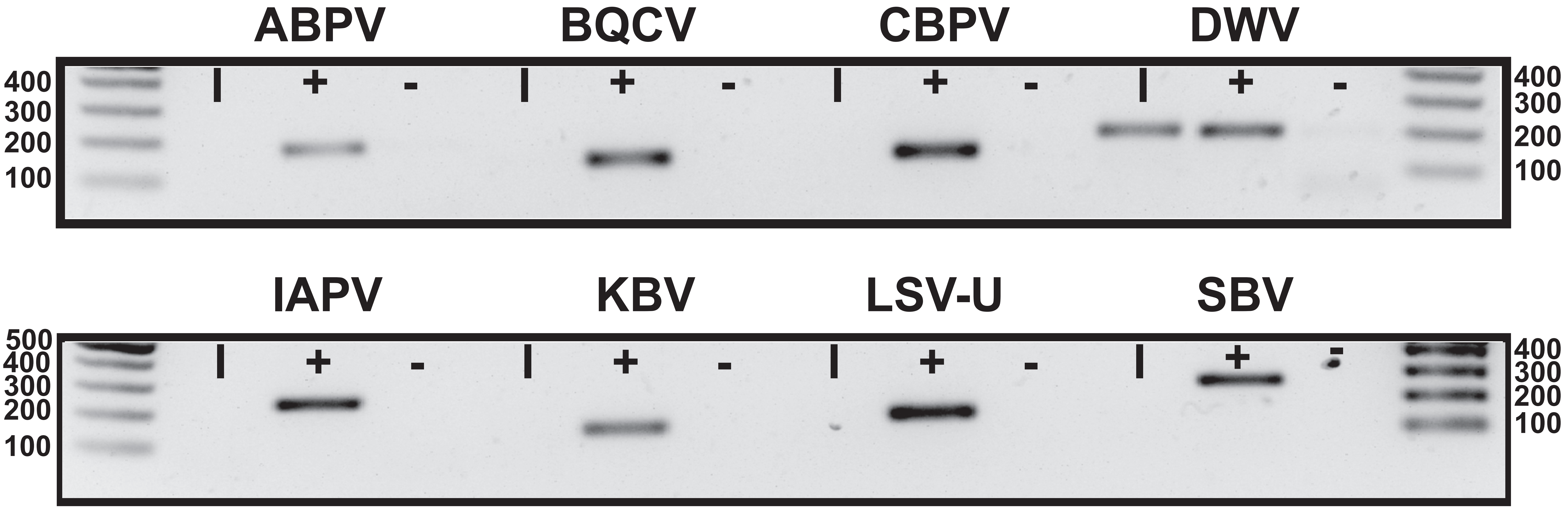

Supplemental Figure S11. Results of DWV inoculum virus testing.
The DWV preparation that was used as inoculum in this study was tested for the presence of contaminating viruses using PCR. RNA was isolated from 100 µL DWV stock and reverse transcribed to synthesize cDNA. Virus-specific PCR was performed to test for the presence of acute bee paralysis virus (ABPV), black queen cell virus (BQCV), chronic bee paralysis virus (CBPV), Israeli acute paralysis virus (IAPV), Kashmir bee virus (KBV), Lake Sinai viruses (i.e., LSV1 and LSV2), sacbrood virus (SBV), and DWV, using primers listed in Supplemental Table S1. The inoculum was confirmed to have DWV and did not have detectable levels any of the other viruses tested.
